# In utero PFAS exposure measured in the umbilical cord blood and child neurodevelopmental outcomes at 24 months: a prospective birth cohort study

**DOI:** 10.1186/s12940-026-01327-2

**Published:** 2026-08-11

**Authors:** Yiming Li, Bingjie Wu, Shiyang Li, Zhichao Yuan, Liu Liu, Yun Huang, Lei Tu, Jun Zhang

**Affiliations:** 1 School of Public Health, Shanghai Jiao Tong University School of Medicine, 227 South Chongqing Road, Shanghai, 200052 China; 2 Ministry of Education–Shanghai Key Laboratory of Children’s Environmental Health, Xinhua Hospital, Shanghai Jiao Tong University School of Medicine, 1665 Kongjiang Road, Shanghai, 200092 China; 3 Tongren Hospital, Shanghai Jiao Tong University School of Medicine, 1111 Xianxia Road, Shanghai, 200336 China

**Keywords:** PFAS, In utero exposure, Cord blood, Neurodevelopment, Birth cohort, Mixture analysis

## Abstract

**Background:**

Prenatal exposure to per- and polyfluoroalkyl substances (PFAS) may be associated with child neurodevelopment, but evidence based on fetal-proximal exposure biomarkers, mixture effects, and domain-specific outcomes remains limited.

**Methods:**

In the Shanghai Birth Cohort (*n* = 1,668 mother-child pairs), we quantified 32 PFAS compounds in umbilical cord blood and assessed neurodevelopment at 24 months using the Bayley Scales of Infant and Toddler Development, Third Edition (BSID-III). Associations were evaluated using adjusted single-pollutant regression models as the primary analyses. Developmental risk defined as BSID-III composite score < 90 was evaluated as a complementary outcome, and weighted quantile sum (WQS) regression was used to evaluate PFAS mixture associations.

**Results:**

Nineteen PFAS compounds had detection rates greater than 65% and were included in association analyses. In adjusted single-pollutant models, several long-chain PFAS were inversely associated with cognitive and language scores. PFNA was associated with lower cognitive (β = −5.39, 95% CI: −7.22, − 3.57) and language scores (β = −3.62, 95% CI: −4.94, − 2.31), as well as higher odds of cognitive developmental risk defined as BSID-III score < 90 (OR = 1.95, 95% CI: 1.40, 2.71). PFUdA, PFTrDA, and PFDoA showed similar inverse associations. The emerging substitute 8:2 Cl-PFESA was also inversely associated with cognitive (β = −1.86, 95% CI: −2.52, − 1.19) and language scores (β = −1.21, 95% CI: −1.68, − 0.73). PFHxA was positively associated with cognitive (β = 1.53), language (β = 0.83), and motor scores (β = 1.15), and PFOA was positively associated with cognitive scores (β = 3.37). In negative-direction WQS regression, the PFAS mixture index was inversely associated with cognitive (β = −4.66, 95% CI: −6.51, − 2.80) and language scores (β = −3.14, 95% CI: −4.65, − 1.63), whereas associations with motor, social-emotional, and adaptive behavior scores were less consistent. No consistent associations were observed for social-emotional or adaptive behavior domains after FDR correction.

**Conclusion:**

Higher cord blood concentrations of selected long-chain PFAS and 8:2 Cl-PFESA were associated with lower cognitive and language performance at 24 months. WQS regression also suggested inverse mixture associations with cognitive and language scores, but these mixture findings should be interpreted cautiously because WQS is directionally constrained and single-pollutant results were not uniformly in the same direction. These findings support continued evaluation of legacy and emerging PFAS in relation to early neurodevelopment.

**Graphical Abstract:**

**Figure:**
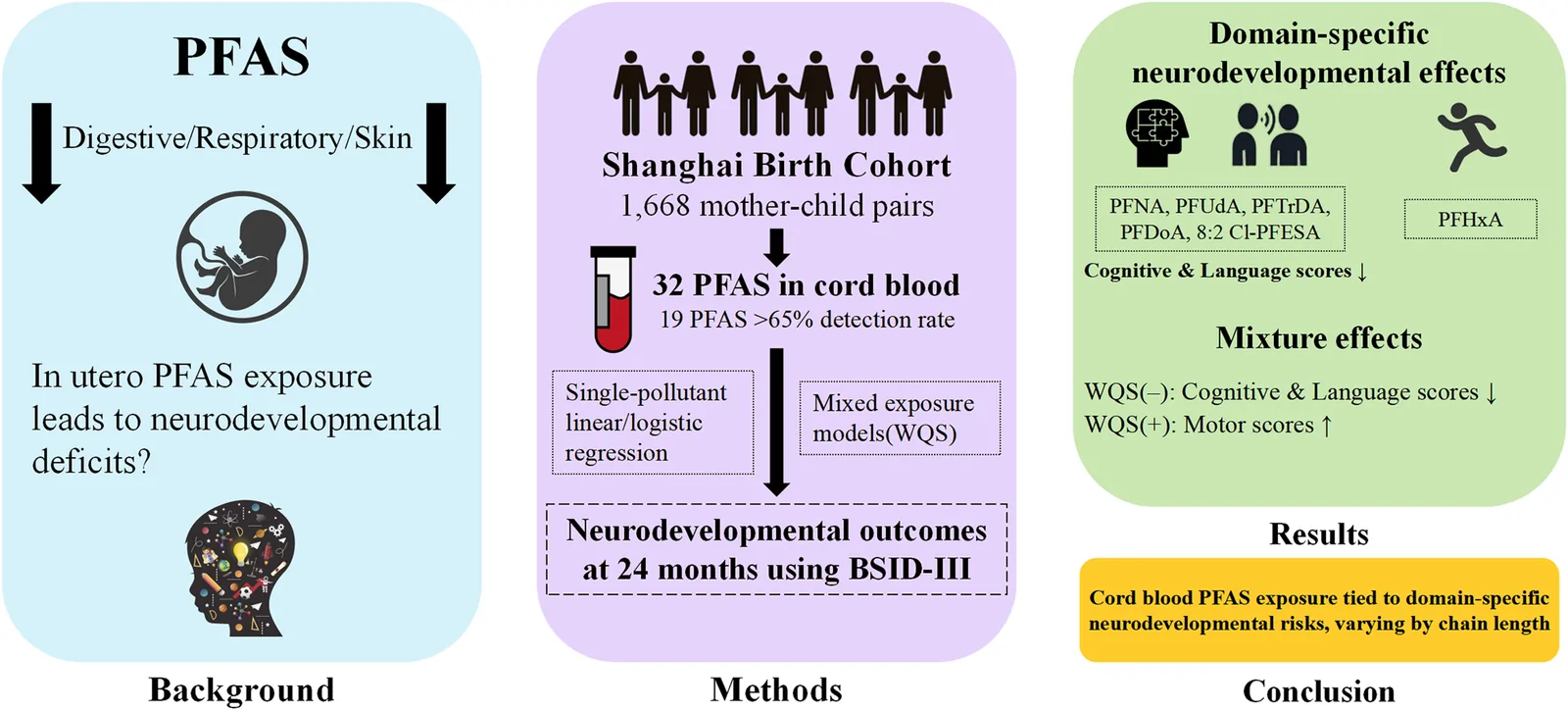

**Supplementary Information:**

The online version contains supplementary material available at https://doi.org/10.1186/s12940-026-01327-2.

## Background

Early neurodevelopment lays the foundation for lifelong health, cognitive capacity, social competence, and economic productivity [1, 2]. The rapid brain growth and synaptogenesis occurring during fetal life and early childhood are critical for the development of cognitive, language, motor, and socioemotional skills [3, 4]. Disruptions during this sensitive window can lead to lasting deficits, with studies linking early-life adversity to impaired educational attainment and socioeconomic outcomes [5, 6]. Environmental factors, particularly exposure to persistent pollutants, have garnered increasing attention due to their potential to interfere with these developmental processes [7]. Among these, per- and polyfluoroalkyl substances (PFAS)—a group of synthetic chemicals ubiquitous in the environment—have emerged as contaminants of concern, especially for their potential impact on neurodevelopment [8, 9].

PFAS are characterized by strong carbon-fluorine bonds, conferring resistance to degradation and unique hydrophobicity and oleophobicity [10]. Widely used since the 1940s in products such as non-stick cookware, firefighting foams, food packaging, and water-repellent textiles, PFAS persist in water, soil, and air, accumulate in human tissues via dietary intake, drinking water, and inhalation [11, 12]. With half-lives ranging from 2 to 7 years for key compounds such as perfluorooctane sulfonate (PFOS), perfluorooctanoic acid (PFOA), and perfluorohexane sulfonate (PFHxS), PFAS bioaccumulate in the blood, liver, and kidneys, posing long-term health risks [13, 14]. Toxicological and epidemiological evidence associates PFAS exposure with reproductive toxicity, immune suppression, liver damage, and endocrine disruption [12]. Notably, in utero exposure—facilitated by PFAS crossing the placental barrier—raises particular concern for fetal brain development [15].

A growing body of epidemiological research has explored the association between prenatal PFAS exposure and child neurodevelopmental outcomes, but findings remain inconsistent. Some studies have reported inverse associations with cognitive function, language development, communication ability, or motor skills [16–18], whereas others have observed null or even positive associations for selected PFAS congeners and developmental domains [19, 20]. Studies examining later neurobehavioral phenotypes, including attention-deficit/hyperactivity disorder (ADHD), autism spectrum disorder (ASD), or autism-related traits, provide additional but conceptually distinct evidence [21–24]. Such outcomes differ from standardized early developmental scores measured in infancy or toddlerhood, and they should not be interpreted as directly interchangeable. Variability across studies may reflect differences in exposure timing, biological matrices, child age at assessment, specific neurodevelopmental domains measured, PFAS congeners included, statistical methods, and potential sex-specific susceptibility [16, 18, 19, 25].

Experimental evidence supports the biological plausibility of PFAS-related neurodevelopmental effects, although behavioral endpoints differ substantially across model organisms. In zebrafish, PFAS studies commonly assess locomotor activity, startle response, exploratory behavior, and neurochemical changes [26]. In rodents, endpoints often include learning and memory, anxiety-like behavior, neurotransmitter signaling, thyroid hormone disruption, and synaptic plasticity markers [15, 27, 28]. In nematode models, neurotoxicity is generally evaluated using endpoints such as locomotion, chemotaxis, neuronal morphology, and molecular pathway alterations rather than anxiety-like behavior in the mammalian sense [15]. These studies suggest that PFAS may interfere with neurodevelopment through multiple mechanisms, including endocrine disruption, altered neurotransmission, oxidative stress, and impaired synaptic plasticity, while also emphasizing the need for careful interpretation across species.

Despite these advances, critical gaps remain in understanding the neurodevelopmental implications of prenatal PFAS exposure. First, exposure assessment often relies on maternal blood or urine, which may not fully reflect fetal-side exposure because of variability in placental transfer efficiency and differences in PFAS pharmacokinetics between maternal and fetal compartments [29]. Umbilical cord blood provides a fetal-proximal biomarker of prenatal exposure near delivery, although it may not capture trimester-specific exposure windows throughout pregnancy. Second, comprehensive evaluations spanning multiple neurodevelopmental domains, including cognitive, language, motor, social-emotional, and adaptive behavior domains, remain limited. Third, emerging PFAS substitutes, such as chlorinated polyfluoroalkyl ether sulfonates (Cl-PFESAs), are insufficiently studied despite their increasing detection in human populations [30]. Finally, the joint effects of correlated PFAS mixtures remain challenging to evaluate, particularly when individual congeners may show associations in opposite directions [31].

This study addresses these gaps by investigating prenatal PFAS exposure and neurodevelopmental outcomes at 24 months using data from the Shanghai Birth Cohort, a prospective birth cohort. By measuring 32 PFAS compounds in umbilical cord blood, we assessed fetal-proximal exposure near delivery. Neurodevelopment was evaluated across five domains—cognitive, language, motor, social-emotional, and adaptive behavior—using the Bayley Scales of Infant and Toddler Development, Third Edition (BSID-III). Single-pollutant models were used as the primary analytic approach to estimate associations for individual PFAS congeners, and WQS regression was applied as a focused secondary approach to evaluate direction-specific PFAS mixture patterns.

## Methods

### Study design and population

This study used data from the Shanghai Birth Cohort (SBC), a prospective cohort study established to investigate early-life determinants of maternal and child health. As previously described [32], the SBC recruited 4,127 pregnant women from six hospitals in Shanghai, China, between 2013 and 2016. After excluding pregnancy losses, stillbirths, and participants lost to postnatal follow-up, 3,692 live-born infants were eligible for postnatal follow-up.

The present analysis included mother-child pairs with both umbilical cord blood samples collected at delivery and neurodevelopmental assessment completed at 24 months of age. Among the eligible live births, 2,381 had available cord blood samples and 3,064 completed the 24-month neurodevelopmental assessment. A total of 1,668 mother-child pairs met both criteria and were included in the final analytical sample (Supplemental Figure S1). The study protocol was approved by the Ethics Committee of Xinhua Hospital affiliated with Shanghai Jiao Tong University School of Medicine. Written informed consent was obtained from all participating mothers at enrollment.

### PFAS exposure assessment

Umbilical cord blood serum was collected within 10 min after delivery using EDTA vacutainers (Becton Dickinson), centrifuged at 3,000 × g for 15 min at 4 °C, aliquoted into pre-labeled cryovials, and stored at − 80 °C until analysis. Cord serum PFAS concentrations were used as fetal-proximal biomarkers of prenatal exposure near delivery. We acknowledge that cord blood reflects fetal-side exposure at birth and may not fully capture trimester-specific exposure windows throughout pregnancy.

Concentrations of 32 per- and polyfluoroalkyl substances (PFAS) were quantified using an optimized ultra-performance liquid chromatography-tandem mass spectrometry (UPLC-MS/MS) method, as described by Ao et al. [33]. Detailed information on PFAS detection limits, detection frequencies, and concentration distributions is provided in Supplemental Table S1. The analytical system consisted of an Agilent 1290 Infinity UPLC coupled to an Agilent 6495 C triple quadrupole mass spectrometer, with detailed chromatographic and mass spectrometric parameters provided in Supplemental Table S2.

To estimate total exposure to major PFAS groups, concentrations of selected structural isomers were summed. Total PFOS was calculated as the sum of n-PFOS, 6 m-PFOS, 3,4,5 m-PFOS, and 1 m-PFOS; total PFHxS was calculated as the sum of n-PFHxS and Br-PFHxS. Individual isomer concentrations were retained for descriptive and supplementary analyses where relevant.

Quality assurance and quality control procedures were implemented throughout sample preparation and instrumental analysis. Method detection limits ranged from 0.002 to 0.016 ng/mL, and limits of quantification ranged from 0.006 to 0.051 ng/mL. Recoveries for spiked serum samples ranged from 80.1% to 116%, with intra-day and inter-day relative standard deviations of 0.9%–12.6% and 0.4%–14.2%, respectively. Calibration curves showed good linearity, with correlation coefficients greater than 0.99, and procedural blanks indicated no detectable contamination. Concentrations below the limit of detection were imputed as LOD/√2 [34].

To ensure reliable exposure estimation, PFAS congeners with detection frequencies greater than 65% were included in the primary regression, correlation, and mixture analyses. PFAS congeners with lower detection frequencies were summarized descriptively but were not included in primary association analyses.

### Neurodevelopmental outcome assessment

Child neurodevelopment was assessed at 24 months of age using the Chinese version of the Bayley Scales of Infant and Toddler Development, Third Edition (BSID-III) [35, 36]. The BSID-III is a standardized instrument designed to evaluate developmental functioning in children aged 1 to 42 months. It includes five domains: cognitive, language, motor, social-emotional, and adaptive behavior. The cognitive, language, and motor domains were assessed directly by trained examiners using structured, age-appropriate tasks. The social-emotional and adaptive behavior domains were evaluated based on parent or caregiver reports [37, 38].

The assessment consisted of tasks of increasing complexity tailored to each domain. For example, in the cognitive domain, children were asked to perform tasks such as inserting shaped blocks into a matching puzzle board [37]. Testing continued until the task difficulty exceeded the child’s developmental capacity. The BSID-III yields raw, scaled, and composite scores. In the present study, composite scores were used as continuous indicators of neurodevelopmental performance, with higher scores indicating better developmental performance.

For binary analyses, developmental risk was defined as a BSID-III composite score < 90. This threshold was selected based on previous epidemiological literature and prior work in this cohort and was used to identify children with below-average developmental performance. We used this cut-off as a risk indicator rather than as a clinical diagnosis of developmental delay.

The Chinese version of the BSID-III has been validated for use in China and has demonstrated acceptable reliability and validity. The cognitive scale showed a Guttman split-half reliability coefficient of 0.803, and correlations with the Gesell Developmental Schedules exceeded 0.80 for most domains [35, 36].

All assessments were conducted by examiners who received standardized training. Testing was performed in a quiet, well-lit, and private room with only the examiner and one parent or caregiver present. Quality assurance was maintained through standardized protocols, periodic site visits by senior staff, and independent checking of calculated scores by quality control personnel [37].

### Covariates

Covariates were selected a priori based on previous literature and a directed acyclic graph (Supplemental Figure S10). The primary fully adjusted models included maternal age, pre-pregnancy body mass index (BMI), parity, maternal education, maternal occupation, self-rated household economic status, child sex, gestational age at delivery, delivery mode, and any breastfeeding duration. Smoking and alcohol consumption during pregnancy were not included because of their very low prevalence in the study population (*n* = 4 for smoking and *n* = 10 for alcohol consumption).

Self-rated household economic status was assessed by questionnaire and categorized as very good, good, budget-conscious, or financially difficult. Any breastfeeding duration was reported by mothers at the 12-month follow-up and categorized as no breastfeeding, < 6 months, or ≥ 6 months. Information on exclusive breastfeeding, breastfeeding intensity, and PFAS concentrations in breast milk was not available. Gestational age at delivery, delivery mode, and any breastfeeding duration were included in the primary models to maintain comparability with previous analyses in this cohort and with prior PFAS-neurodevelopment studies. However, because these variables may also be conceptualized as potential mediators, downstream factors, or postnatal determinants of neurodevelopment, we additionally conducted reduced-model sensitivity analyses excluding gestational age at delivery, delivery mode, and any breastfeeding duration.

### Statistical analysis

#### Data preprocessing and descriptive statistics

Maternal and child characteristics were summarized using means and standard deviations for continuous variables and frequencies and percentages for categorical variables. PFAS concentrations were right-skewed and therefore summarized as medians and interquartile ranges. PFAS concentrations were log10-transformed for regression analyses to reduce skewness and limit the influence of extreme values [39]. Regression coefficients for continuous PFAS exposures therefore represent differences in BSID-III scores per log10-unit increase in cord serum PFAS concentration, corresponding approximately to a ten-fold increase in exposure.

Only PFAS congeners with detection frequencies greater than 65% were included in association analyses. Concentrations below the limit of detection (LOD) were imputed as LOD/√2, using a standard substitution approach for left-censored environmental exposure data [40]. Spearman correlation coefficients were calculated to evaluate correlations among included PFAS congeners. The Wilcoxon rank-sum test was used to compare PFAS concentrations by child sex.

Missing covariate data were addressed using multiple imputation by fully conditional specification [41]. The imputation model included all covariates used in the analytic models: maternal age, pre-pregnancy BMI, parity, maternal education, maternal occupation, self-rated household economic status, gestational age at delivery, child sex, delivery mode, and any breastfeeding duration. PFAS concentrations and BSID-III composite scores were also included as predictors to improve compatibility between the imputation and analytic models, but PFAS exposures and BSID-III outcomes themselves were not imputed. Values below the limit of detection for PFAS were replaced with LOD/√2 before imputation and were not treated as missing values. Twenty imputed datasets were generated, and convergence of the imputation algorithm was evaluated by visual inspection of trace plots. Continuous variables were imputed using predictive mean matching, binary variables using logistic regression, unordered categorical variables using multinomial logistic regression, and ordered categorical variables using proportional odds models, as appropriate. Regression models were fitted separately within each imputed dataset, and estimates were pooled using Rubin’s rules. The proportions of missingness for all imputed covariates are reported in Supplemental Table S9. PFAS values exceeding three times the 75th percentile were retained in the primary analyses but excluded in sensitivity analyses to evaluate the influence of extreme exposure values.

#### Primary single-pollutant analyses

Single-pollutant regression models were prespecified as the primary analyses to estimate associations between individual PFAS congeners and neurodevelopmental outcomes. PFAS concentrations were modeled primarily as log10-transformed continuous variables because this metric retains exposure information, avoids arbitrary cut points, and allows coefficients to be interpreted as differences in BSID-III scores per approximately ten-fold increase in PFAS concentration. For continuous outcomes, multivariable linear regression models were used to assess associations between each log10-transformed PFAS congener and BSID-III composite scores in the cognitive, language, motor, social-emotional, and adaptive behavior domains. Each model included one PFAS congener at a time and was adjusted for the covariates listed above. Crude associations from unadjusted linear regression models are presented in Supplemental Table S3 for comparison.

Quartile-based analyses were conducted as supplementary analyses to evaluate whether the primary findings were robust to exposure categorization and to describe broad exposure gradients. PFAS concentrations were categorized into quartiles, with the lowest quartile as the reference group. Linear trends across quartiles were assessed by assigning the median value of each quartile and modeling it as a continuous variable. Quartile-based results are presented in Supplemental Table S4.

As complementary outcome analyses, multivariable logistic regression models were used to estimate odds ratios and 95% confidence intervals for developmental risk, defined as BSID-III composite score < 90, compared with scores ≥ 90. These models used the same continuous log10-transformed PFAS exposure metrics and covariate adjustment strategy as the primary linear regression models. The false discovery rate (FDR) method was applied to account for multiple testing across PFAS congeners for each outcome domain, unless otherwise specified, and FDR-adjusted p-values < 0.05 were considered statistically significant [42].

#### Non-linear exposure-response analyses

Restricted cubic spline models were used as supplementary analyses to explore whether associations between individual PFAS congeners and BSID-III outcomes departed from linearity. Five knots were placed at the 5th, 27.5th, 50th, 72.5th, and 95th percentiles of each PFAS distribution [43]. These analyses were interpreted as exploratory checks of the linearity assumption and are presented in the Supplementary Materials.

#### Mixture analyses

Because PFAS congeners are moderately to highly correlated and individuals are exposed to PFAS mixtures, weighted quantile sum (WQS) regression was selected as the main mixture approach to evaluate direction-specific associations of the PFAS mixture with BSID-III outcomes. WQS creates a weighted index from quantiled exposures and assigns each component a weight that reflects its relative contribution to the mixture-outcome association under a specified directional constraint [44, 45].

We selected WQS because it has been used in previous Shanghai Birth Cohort PFAS analyses to summarize correlated prenatal PFAS exposures and identify influential congeners in a mixture context [46]. WQS is a unidirectional mixture method in which all components are assumed to contribute to the mixture index in the same specified direction within a given model. Therefore, we used the overall study hypothesis and the single-pollutant results to guide the directionality of WQS analyses. The negative-direction WQS model was presented in the main manuscript because it corresponded to the a priori concern that higher PFAS exposure may be associated with poorer neurodevelopment and was consistent with the primary inverse single-pollutant findings for several long-chain PFAS and 8:2 Cl-PFESA. Positive-direction WQS results were provided in the Supplementary Materials because they represented exploratory patterns that were less consistent with the primary adverse neurodevelopment hypothesis. Both negative- and positive-direction WQS results were interpreted cautiously because the estimates and weights depend on the imposed directional constraint.

#### Sensitivity analyses

Several sensitivity analyses were conducted to assess the robustness of the findings. First, baseline characteristics were compared between participants included in and excluded from the final analytical sample to evaluate potential selection bias. Second, single-pollutant linear and logistic regression models were repeated after excluding PFAS values exceeding three times the 75th percentile to assess the influence of extreme exposure values (Supplemental Table S5). Third, to address concerns about potential overadjustment, we repeated the main single-pollutant linear and logistic regression analyses using reduced covariate models that excluded gestational age at delivery, delivery mode, and any breastfeeding duration (Supplemental Tables S6 and S7). In the reduced logistic models, maternal occupation was additionally omitted because sparse categories led to model instability. Finally, positive-direction WQS regression was retained as a supplementary sensitivity analysis of mixture patterns (Supplemental Table S8 and Supplemental Figure S8).

All statistical analyses were performed using R statistical software, version 4.5.1 (R Core Team). Two-sided p-values < 0.05 were considered statistically significant unless otherwise specified.

## Results

### Participant characteristics

A total of 1,668 mother-child pairs from the Shanghai Birth Cohort were included in the final analysis. The mean maternal age was 28.6 years (SD = 3.6), and the mean pre-pregnancy body mass index (BMI) was 21.4 kg/m² (SD = 3.1). The mean gestational age at delivery was 38.9 weeks (SD = 1.5). Most mothers were nulliparous (*n* = 1,237, 74.2%) and had a college education or higher (*n* = 1,317, 79.0%). The most common maternal occupations were office worker (29.0%) and professional/technical worker (23.4%). Self-rated household economic status was most frequently reported as good (67.4%) or very good (15.2%). Among the children, 840 (50.4%) were male and 806 (48.3%) were female. Vaginal delivery occurred in 809 births (48.5%), cesarean delivery in 753 births (45.1%), and forceps delivery in 34 births (2.0%). Any breastfeeding for ≥ 6 months was reported for 1,259 infants (75.5%), while 277 infants (16.6%) were breastfed for < 6 months. Missing covariate values before multiple imputation are shown in Table 1 and summarized in Supplemental Table S9. Detailed demographic and clinical characteristics are shown in Table 1.

**Table 1:** Demographic characteristics of study participants in the Shanghai birth cohort

Characteristics	Category	*n* (%)
Maternal Characteristics		
Maternal age (years)	Mean ± SD	28.6 ± 3.6
Pre-pregnancy BMI (kg/m²)	Mean ± SD	21.4 ± 3.1
Gestational weeks	Mean ± SD	38.9 ± 1.5
Parity	0	1237 (74.2)
	≥ 1	202 (12.1)
	Missing before multiple imputation	229 (13.7)
Maternal education	≤High school	123 (7.4)
	≥College	1317 (79.0)
	Missing before multiple imputation	228 (13.7)
Occupation	Worker	18 (1.1)
	Farmer	1 (0.1)
	Professional/Technical	391 (23.4)
	Office Worker	483 (29.0)
	Service/Sales	229 (13.7)
	Missing before multiple imputation	546 (32.7)
Self-rated household economic status	Very good	254 (15.2)
	Good	1124 (67.4)
	Budget-conscious	14 (0.8)
	Financially difficult	2 (0.1)
	Missing before multiple imputation	274 (16.4)
Child Characteristics		
Child sex	Female	806 (48.3)
	Male	840 (50.4)
	Missing before multiple imputation	22 (1.3)
Delivery mode	Vaginal	809 (48.5)
	Cesarean	753 (45.1)
	Forceps	34 (2.0)
	Missing before multiple imputation	72 (4.3)
Any breastfeeding duration	No breastfeeding	40 (2.4)
	< 6 months	277 (16.6)
	≥ 6 months	1259 (75.5)
	Missing before multiple imputation	92 (5.5)
Total participants		1668 (100)

Values are presented as mean ± Standard deviation (SD) for continuous variables (maternal age, pre-pregnancy BMI, gestational weeks) and as number (percentage) for categorical variables. Missing categories represent values missing before multiple imputation and were not treated as analytical categories in regression models. *BMI* Body Mass Index

### PFAS concentrations in cord blood

Concentrations of 32 PFAS were quantified in cord blood serum. Detection frequencies varied across congeners, and 19 PFAS with detection frequencies greater than 65% were included in the primary association analyses. PFAS concentrations were right-skewed and are summarized as medians and interquartile ranges in Supplemental Table S1. Among the included congeners, several legacy and emerging PFAS were frequently detected, including 6:2 Cl-PFESA, PFNA, PFOA, PFOS isomers, and PFUdA. For example, PFNA had a median concentration of 0.660 ng/mL (IQR: 0.469–0.967), and n-PFOS had a median concentration of 1.971 ng/mL (IQR: 1.323–2.881). PFOA showed the highest median concentration among measured PFAS (median: 6.242 ng/mL). Wilcoxon rank-sum tests showed no statistically significant differences in PFAS concentrations between male and female infants. Detailed analytical parameters and concentration distributions are provided in Supplemental Tables S1 and S2.

### Inter-congener correlations of PFAS in cord blood

Spearman correlation coefficients among PFAS congeners measured in cord blood serum are shown in Supplemental Figure S2. Overall, most included PFAS congeners showed positive correlations of varying magnitude, particularly among structural isomers and long-chain perfluoroalkyl acids. These correlation patterns suggested shared exposure sources or similar pharmacokinetic properties, supporting the use of WQS regression as a focused secondary mixture approach. Correlations involving congeners with low detection frequencies were not calculated or were interpreted cautiously.

### Associations between cord blood PFAS and BSID-III scores

Unadjusted associations between cord blood PFAS concentrations and BSID-III scores are presented in Supplemental Table S3. In covariate-adjusted single-pollutant models, several long-chain perfluoroalkyl carboxylic acids showed inverse associations with cognitive and language scores (Table 2). PFNA was associated with lower cognitive scores (β= -5.39, 95%CI: -7.22, -3.57) and lower language scores (β= -3.62, 95%CI: -4.94, -2.31). Similar inverse associations were observed for PFUdA with cognitive (β= -5.04, 95%CI: -6.69, -3.38) and language scores (β= -3.01, 95%CI: -4.20, -1.81), and for PFTrDA with cognitive (β= -4.07, 95%CI: -5.78, -2.37) and language scores (β= -2.31, 95%CI: -3.54, -1.09). PFDoA was also inversely associated with cognitive (β= -1.91, 95%CI: -3.16, -0.66) and language scores (β= -1.39, 95%CI: -2.29, -0.49).

**Table 2:** Adjusted linear regression coefficients (β) and 95% confidence Intervals for BSID-III scores associated with cord blood PFAS concentrations in single-pollutant models (*n* = 1,668)

PFAS	β (95%CI) *n* = 1668				
	cognitive scores	language scores	motor scores	Socio-emotional scores	Adaptive behavior scores
6:2 Cl-PFESA	-0.77 (-2.36,0.83)	-0.74 (-1.89,0.41)	0.50 (-0.62,1.62)	-0.81 (-2.19,0.57)	0.86 (-0.63,2.36)
8:2 Cl-PFESA	**-1.86 (-2.52**,-1.19)^***^	**-1.21 (-1.68**,-0.73)^***^	-0.50 (-0.97,-0.03)	0.26 (-0.32,0.83)	0.84 (0.21,1.47)
FOSA	0.41 (-0.85,1.66)	0.17 (-0.73,1.07)	0.50 (-0.38,1.38)	-0.04 (-1.12,1.04)	0.38 (-0.80,1.56)
HFPO-DA	-0.27 (-0.99,0.45)	-0.59 (-1.10,-0.07)	-0.16 (-0.66,0.35)	0.77 (0.15,1.39)	0.35 (-0.32,1.03)
PFBA	0.67 (-1.06,2.39)	-0.07 (-1.31,1.17)	1.25 (0.05,2.46)	-0.78 (-2.29,0.73)	1.70 (0.06,3.35)
PFBS	-0.10 (-1.14,0.94)	0.00 (-0.75,0.75)	0.53 (-0.20,1.25)	0.24 (-0.66,1.14)	0.53 (-0.44,1.51)
PFDA	0.28 (-0.93,1.49)	-0.15 (-1.02,0.73)	0.61 (-0.24,1.46)	-0.04 (-1.08,1.01)	0.31 (-0.82,1.44)
PFDoA	**-1.91 (-3.16**,-0.66)^**^	**-1.39 (-2.29**,-0.49)^**^	-0.50 (-1.38,0.37)	0.42 (-0.66,1.50)	1.24 (0.07,2.42)
PFHpA	**1.02 (0.24**,1.80)^*^	0.33 (-0.23,0.89)	0.56 (0.02,1.11)	0.30 (-0.38,0.97)	-0.05 (-0.78,0.68)
PFHpS	-0.16 (-2.17,1.84)	-0.86 (-2.31,0.58)	-0.01 (-1.42,1.39)	-0.45 (-2.18,1.28)	1.57 (-0.31,3.45)
PFHxA	**1.53 (0.74**,2.32)^***^	**0.83 (0.26**,1.39)^*^	**1.15 (0.59**,1.70)^***^	0.56 (-0.12,1.24)	0.44 (-0.30,1.18)
PFHxS(Total)	2.29 (0.29,4.30)	-0.18 (-1.62,1.27)	1.24 (-0.16,2.65)	-0.13 (-1.87,1.61)	1.15 (-0.75,3.05)
PFNA	**-5.39 (-7.22**,-3.57)^***^	**-3.62 (-4.94**,-2.31)^***^	-1.33 (-2.61,-0.04)	-0.82 (-2.41,0.77)	1.95 (0.22,3.68)
PFOA	**3.37 (1.18**,5.56)^**^	0.42 (-1.16,2.00)	1.14 (-0.40,2.67)	-0.73 (-2.63,1.16)	0.34 (-1.73,2.41)
PFOS(Total)	-0.48 (-2.26,1.30)	-0.69 (-1.97,0.59)	0.42 (-0.83,1.67)	-0.40 (-1.94,1.14)	1.41 (-0.27,3.09)
PFPeS	-1.30 (-2.61,0.01)	**-1.81 (-2.75**,-0.87)^***^	-1.08 (-2.00,-0.17)	0.72 (-0.41,1.84)	0.64 (-0.58,1.86)
PFTeDA	-0.98 (-1.87,-0.08)	-0.60 (-1.25,0.04)	-0.02 (-0.64,0.61)	-0.38 (-1.15,0.39)	0.10 (-0.74,0.94)
PFTrDA	**-4.07 (-5.78**,-2.37)^***^	**-2.31 (-3.54**,-1.09)^***^	-0.18 (-1.37,1.02)	-0.41 (-1.89,1.07)	0.82 (-0.79,2.44)
PFUdA	**-5.04 (-6.69**,-3.38)^***^	**-3.01 (-4.20**,-1.81)^***^	-0.96 (-2.13,0.21)	-0.57 (-2.02,0.87)	1.57 (-0.00,3.14)

1. Single-pollutant models included one PFAS compound at a time, with log₁₀-transformed concentrations (per log₁₀-unit increase, ng/mL), adjusted for maternal age, pre-pregnancy BMI, gestational age, parity, education, occupation, self-rated household economic status, child sex, delivery mode, and any breastfeeding duration

2. *β*  Regression coefficient, *CI*  Confidence interval, *BSID-III* Bayley Scales of Infant and Toddler Development, Third Edition

3. Bolded values indicate significant associations after False Discovery Rate (FDR) correction; significance levels: *p < 0.05, **p < 0.01, ***p < 0.001

4. Full compound names are provided in Supplemental Table S1

The emerging substitute 8:2 Cl-PFESA was inversely associated with cognitive (β= -1.86, 95% CI: -2.52, -1.19) and language scores (β= -1.21, 95%CI: -1.68, -0.73). In contrast, PFHxA showed positive associations with cognitive (β = 1.53, 95%CI: 0.74, 2.32), language (β = 0.83, 95%CI: 0.26, 1.39), and motor scores (β = 1.15, 95%CI: 0.59, 1.70), while PFOA was positively associated with cognitive scores (β = 3.37, 95%CI: 1.18, 5.56). No associations for social-emotional or adaptive behavior scores remained statistically significant after FDR correction.

Quartile-based analyses were generally consistent with the continuous single-pollutant models for the principal cognitive and language findings, although exposure-response patterns were not uniformly monotonic across all PFAS congeners and domains (Supplemental Table S4).

### Associations between cord Blood PFAS and developmental risk

Using BSID-III composite score < 90 as an indicator of developmental risk, logistic regression analyses showed patterns broadly consistent with the continuous-score analyses (Table 3). Long-chain PFCAs were associated with higher odds of below-average cognitive and language performance. PFNA was associated with higher odds of cognitive developmental risk (OR = 1.95, 95% CI: 1.40, 2.71) and language developmental risk (OR = 1.72, 95% CI: 1.41, 2.11). PFUdA was associated with higher odds of cognitive (OR = 1.73, 95% CI: 1.28, 2.36) and language developmental risk (OR = 1.57, 95% CI: 1.30, 1.89), and PFTrDA was associated with higher odds of cognitive (OR = 1.53, 95% CI: 1.11, 2.13) and language developmental risk (OR = 1.41, 95% CI: 1.17, 1.71). PFDoA was associated with higher odds of language developmental risk (OR = 1.25, 95% CI: 1.09, 1.43).

**Table 3:** Complementary analyses of developmental risk defined as BSID-III composite score < 90 associated with cord blood PFAS concentrations

PFAS	OR (95%CI)				
	Cognitive developmental risk	Language developmental risk	Motor developmental risk	Social-emotional developmental risk	Adaptive developmental risk
6:2 Cl-PFESA	1.22 (0.91,1.63)	1.10 (0.93,1.31)	0.83 (0.60,1.14)	1.10 (0.90,1.35)	0.89 (0.74,1.07)
8:2 Cl-PFESA	**1.25 (1.09**,**1.46)**	**1.18 (1.09**,**1.27)**	1.10 (0.95,1.28)	0.99 (0.91,1.07)	0.91 (0.85,0.98)
FOSA	0.86 (0.70,1.07)	0.98 (0.85,1.12)	0.77 (0.61,0.98)	0.98 (0.84,1.15)	1.00 (0.87,1.16)
HFPO-DA	1.13 (1.00,1.29)	1.11 (1.02,1.20)	1.07 (0.93,1.24)	0.97 (0.88,1.06)	0.95 (0.87,1.03)
PFBA	0.82 (0.59,1.14)	1.01 (0.83,1.22)	1.00 (0.70,1.42)	1.01 (0.81,1.26)	0.78 (0.63,0.96)
PFBS	1.07 (0.88,1.32)	1.02 (0.91,1.14)	0.99 (0.80,1.23)	0.99 (0.87,1.13)	0.90 (0.80,1.01)
PFDA	0.80 (0.66,0.98)	1.03 (0.90,1.17)	**0.74 (0.60**,**0.91)**	0.93 (0.80,1.09)	0.93 (0.81,1.07)
PFDoA	1.01 (0.83,1.23)	**1.25 (1.09**,**1.43)**	0.95 (0.75,1.20)	0.87 (0.74,1.02)	**0.81 (0.69**,**0.94)**
PFHpA	0.87 (0.77,0.99)	0.96 (0.88,1.04)	0.89 (0.77,1.03)	1.02 (0.92,1.12)	0.95 (0.87,1.03)
PFHpS	1.33 (0.92,1.91)	1.22 (0.99,1.52)	0.98 (0.66,1.45)	1.06 (0.82,1.36)	0.79 (0.62,0.99)
PFHxA	0.91 (0.79,1.05)	0.93 (0.85,1.01)	0.89 (0.76,1.04)	0.99 (0.89,1.09)	0.94 (0.86,1.03)
PFHxS(Total)	0.95 (0.65,1.37)	1.20 (0.97,1.49)	0.76 (0.49,1.15)	1.02 (0.79,1.32)	0.83 (0.65,1.05)
PFNA	**1.95 (1.40**,**2.71)**	**1.72 (1.41**,**2.11)**	1.05 (0.74,1.51)	1.02 (0.81,1.29)	0.79 (0.64,0.98)
PFOA	0.85 (0.58,1.27)	1.03 (0.82,1.31)	0.77 (0.50,1.19)	0.99 (0.74,1.31)	0.90 (0.70,1.17)
PFOS(Total)	1.20 (0.87,1.66)	1.14 (0.94,1.38)	0.81 (0.56,1.15)	1.05 (0.84,1.31)	0.86 (0.69,1.05)
PFPeS	1.27 (1.00,1.62)	**1.40 (1.21**,**1.63)**	1.21 (0.93,1.58)	0.94 (0.80,1.10)	0.94 (0.81,1.09)
PFTeDA	1.01 (0.86,1.19)	1.00 (0.91,1.10)	0.98 (0.82,1.17)	1.11 (0.99,1.25)	0.99 (0.89,1.10)
PFTrDA	1.53 (1.11,2.13)	**1.41 (1.17**,**1.71)**	0.84 (0.61,1.18)	1.00 (0.81,1.24)	0.91 (0.74,1.11)
PFUdA	**1.73 (1.28**,**2.36)**	**1.57 (1.30**,**1.89)**	0.98 (0.71,1.36)	1.00 (0.81,1.24)	0.81 (0.66,0.98)

1. *OR* Odds Ratio, *CI*  Confidence Interval, *BSID-III* Bayley Scales of Infant and Toddler Development, Third Edition

2. Developmental risk was defined as a BSID-III composite score < 90; the reference group was BSID-III score ≥ 90

3. All models adjusted for maternal age, pre-pregnancy BMI, gestational age, parity, education, occupation, self-rated household economic status, child sex, delivery mode, and any breastfeeding duration

4. Boldface indicates associations that remained significant after false discovery rate (FDR) correction (q < 0.05)

The replacement compound 8:2 Cl-PFESA was also associated with higher odds of cognitive developmental risk (OR = 1.25, 95% CI: 1.09, 1.46) and language developmental risk (OR = 1.18, 95% CI: 1.09, 1.27). For language, elevated odds were additionally observed for PFPeS (OR = 1.40, 95% CI: 1.21, 1.63) and HFPO-DA (OR = 1.11, 95% CI: 1.02, 1.20). Associations for social-emotional developmental risk were small and did not remain significant after FDR correction.

### Mixture effects

WQS regression was conducted to evaluate direction-specific mixture patterns across the correlated PFAS congeners. As shown in Table 4, in the negative-direction WQS model, the PFAS mixture index was inversely associated with cognitive scores (β = −4.66, 95% CI: −6.51, − 2.80; *p* < 0.01) and language scores (β = −3.14, 95% CI: −4.65, − 1.63; *p* < 0.01). Associations were weaker or inconsistent for motor (β = −1.38, 95% CI: −2.80, 0.03; *p* = 0.06), social-emotional (β = −0.43, 95% CI: −2.10, 1.25; *p* = 0.62), and adaptive behavior scores (β = 1.84, 95% CI: 0.00, 3.68; *p* > 0.05). Component weights for the negative-direction WQS models are shown in Supplemental Figure S9. Positive-direction WQS results, presented as supplementary sensitivity analyses, are provided in Supplemental Table S8 and Supplemental Figure S8.

**Table 4:** Negative-direction Weighted Quantile Sum (WQS) regression results for prenatal PFAS mixtures and neurodevelopmental outcomes at 24 months

Method	Outcome Domain	Effect Estimate (β) (95% CI)	*P*-value
WQS regression (negative-direction constraint)	cognitive scores	-4.66 (-6.51, -2.80)	< 0.01
	Language scores	-3.14 (-4.65, -1.63)	< 0.01
	Motor scores	-1.38 (-2.80, 0.03)	0.06
	Social-emotional scores	-0.43 (-2.10, 1.25)	0.62
	Adaptive behavior scores	1.84 (0.00, 3.68)	> 0.05

1. WQS results are expressed as differences in BSID-III scores per one-unit increase in the WQS index, corresponding to a one-quartile increase in the weighted PFAS mixture index. Component weights for the negative-direction WQS models are shown in Supplemental Figure S9. Positive-direction WQS results are provided in Supplemental Table S8 and Supplemental Figure S8

2. Statistically significant associations (*p* < 0.05) are shown in bold

### Sensitivity analyses

After excluding PFAS values greater than three times the 75th percentile, the main patterns of association were generally unchanged (Supplemental Table S5). Long-chain PFCAs, including PFNA, PFUdA, PFTrDA, and PFDoA, as well as 8:2 Cl-PFESA, remained inversely associated with cognitive and language scores, whereas PFHxA and PFOA continued to show positive associations with selected BSID-III scores.

To address concerns about potential overadjustment, we repeated the single-pollutant linear and logistic regression models using reduced covariate models that excluded gestational age at delivery, delivery mode, and any breastfeeding duration. The main findings were materially unchanged in both reduced linear and reduced logistic models (Supplemental Tables S6 and S7). For example, inverse associations with cognitive and language scores persisted for PFNA, PFUdA, PFTrDA, and 8:2 Cl-PFESA, and similar patterns were observed for developmental risk. These findings indicate that the main results were robust to excluding potential mediators or postnatal factors from the covariate set.

## Discussion

This prospective study within the Shanghai Birth Cohort investigated associations between prenatal exposure to 32 PFAS measured in umbilical cord blood and neurodevelopmental outcomes at 24 months, assessed using the BSID-III. The most consistent associations were observed for cognitive and language outcomes. In single-pollutant models, several long-chain PFCAs, including PFNA, PFUdA, PFTrDA, and PFDoA, were associated with lower cognitive and language scores, and 8:2 Cl-PFESA showed similar inverse associations. PFHxA and PFOA showed positive associations with selected BSID-III domains, but these unexpected findings should be interpreted cautiously. These results were by and large confirmed by the PFAS mixture analysis using WQS.

The interpretation of these effect estimates requires context. BSID-III composite scores are standardized developmental scores, and a 1–2-point difference may be modest at the individual clinical level [37, 38]. However, the estimates observed for several long-chain PFCAs, particularly PFNA and PFUdA, correspond to approximately one-third of a standard deviation if evaluated against the conventional 15-point standard deviation of Bayley composite scores. Such differences should not be interpreted as individual clinical diagnoses. Nevertheless, from a population-health perspective, a small leftward shift in developmental score distributions may increase the proportion of children falling below developmental-risk thresholds, particularly when exposure is widespread. Therefore, the BSID-III < 90 threshold in this study should be understood as an indicator of below-average developmental performance or developmental risk, rather than as a clinical diagnosis of developmental delay.

Our findings for long-chain PFAS are generally consistent with prior epidemiological evidence, although the literature remains heterogeneous. For PFNA, adverse associations with early cognitive and language outcomes are compatible with studies linking prenatal PFAS exposure to autism-related traits or less favorable neurodevelopmental performance [23]. Additional evidence from the MIREC cohort suggested that prenatal exposure to legacy PFAS mixtures may be associated with lower performance IQ in preschool-aged children, particularly among boys [25]. Previous studies have also reported associations between prenatal PFAS exposure and language or communication development, although the direction and magnitude of these associations varied across cohorts [17, 18]. In contrast, other studies have reported null or positive findings for selected PFAS or domains [19, 20]. These mixed results likely reflect differences in child age at assessment, neurodevelopmental instruments, exposure windows, biological matrices, PFAS panels, and population characteristics. Compared with studies of ADHD, ASD, or later behavioral traits [21, 23, 24], our study focused on standardized early developmental performance at 24 months; therefore, direct comparison across outcome types should be made cautiously.

The inverse associations observed for 8:2 Cl-PFESA are noteworthy because epidemiological evidence on emerging PFAS substitutes and child neurodevelopment remains limited. Experimental studies suggest that F-53B and related chlorinated ether sulfonates may affect fetal neurodevelopment through placental and thyroid hormone disruption, altered neurotransmitter signaling, or changes in synaptic plasticity markers [28, 47]. Our findings extend this concern to a human prospective birth cohort and suggest that emerging PFAS substitutes warrant attention in developmental epidemiology. Nevertheless, because evidence on Cl-PFESAs and early neurodevelopment is still sparse, these associations require replication in independent cohorts with repeated exposure assessment and longer follow-up.

The positive associations observed for PFHxA and PFOA should be interpreted cautiously and should not be regarded as evidence of neurodevelopmental benefit. Existing studies often do not evaluate PFHxA separately or report consistent evidence of beneficial associations [12, 20]. PFOA findings have also varied across studies; for example, Xie et al. reported associations between prenatal PFOA exposure and attention problems at age 6 years in the Shanghai-Minhang Birth Cohort [48], which differs from the positive association with cognition observed at 24 months in the present study. Several explanations may contribute to these unexpected positive associations. Residual socioeconomic or lifestyle-related confounding may remain even after adjustment for maternal education and self-rated household economic status, because some PFAS exposure sources may correlate with diet, consumer product use, healthcare access, or early developmental stimulation. Differences in toxicokinetics, placental transfer efficiency, and exposure sources across PFAS congeners may also contribute to compound-specific patterns [49, 50]. In addition, non-monotonic dose-response relationships have been described for endocrine-disrupting chemicals [51], and some positive findings may also reflect chance or the influence of correlated PFAS congeners with associations in opposing directions. Therefore, PFHxA and PFOA findings should be considered unexpected and hypothesis-generating rather than protective.

The present cord-blood analysis and our prior maternal-blood study in the same cohort [52] provide complementary evidence. Both studies used the same BSID-III assessment at 24 months and identified inverse associations between long-chain PFCAs and cognition or language. However, the two studies differ in exposure matrix and timing: maternal plasma was collected in early pregnancy in the prior study, whereas cord serum in the present analysis reflects fetal-side exposure near delivery. Maternal blood and cord blood are related but not interchangeable, because placental transfer varies with chain length, functional group, protein binding, and maternal-fetal physiology [29, 49, 50]. Maternal plasma in early pregnancy may better represent exposure during neurogenesis, neuronal migration, and maternal thyroid-dependent stages, whereas cord blood may better approximate fetal exposure accumulated by late gestation, closer to synaptogenesis and early myelination. Differences in PFOS, PFOA, PFHxA, and adaptive behavior findings across the two studies may therefore reflect exposure timing, matrix-specific transfer patterns, and differences in the PFAS panel, especially the inclusion of emerging compounds such as 8:2 Cl-PFESA in the present study.

WQS regression provided secondary evidence on correlated PFAS mixtures but requires cautious interpretation. This approach is useful for summarizing highly correlated exposures into a single mixture index and for identifying components with relatively larger weights, and it has previously been applied in the Shanghai Birth Cohort to evaluate prenatal PFAS mixtures [46]. We presented the negative-direction WQS model in the main text because it was aligned with the primary adverse neurodevelopmental hypothesis and with the most consistent single-pollutant findings for long-chain PFAS and 8:2 Cl-PFESA. However, WQS is directionally constrained: within a given model, all mixture components are assumed to contribute in the same specified direction. This assumption is important in the present study because single-pollutant models also showed unexpected positive associations for PFHxA and PFOA. Positive-direction WQS analyses, presented in the Supplementary Materials, were therefore interpreted as exploratory sensitivity analyses. Overall, we interpret the WQS findings as direction-specific prioritization and sensitivity evidence rather than as a single homogeneous overall PFAS mixture effect. Component weights should not be viewed as independent causal effects of individual PFAS congeners, and the mixture results should be considered secondary to the single-pollutant findings.

Breastfeeding requires careful interpretation in this setting. Any breastfeeding duration may reflect beneficial postnatal nutrition, maternal-child interaction, and social bonding, all of which are relevant to early neurodevelopment. At the same time, breastfeeding may also represent an important route of postnatal PFAS transfer to the infant, particularly during the first year of life [12, 50]. In addition, breastfeeding duration may be influenced by maternal or perinatal factors related to prenatal PFAS exposure and child development. Therefore, adjustment for breastfeeding duration may control for some postnatal developmental influences but may also partially adjust for pathways related to postnatal PFAS exposure or mediation. The similar results observed in reduced-covariate models excluding breastfeeding duration suggest that the main cognitive and language findings were not driven solely by this adjustment decision. However, these analyses cannot distinguish prenatal PFAS effects from PFAS exposure transmitted through breast milk, nor can they determine whether breastfeeding lies on a causal pathway. Future studies with breast-milk PFAS measurements, infant postnatal biomarkers, and more detailed feeding information are needed to clarify these pathways.

Mechanistically, the observed inverse associations for long-chain PFCAs and 8:2 Cl-PFESA are biologically plausible. PFAS may interfere with neurodevelopment through disruption of thyroid hormone homeostasis, placental function, neurotransmitter signaling, oxidative stress, and synaptic plasticity [15, 27, 28, 47]. These pathways are highly relevant during fetal and early postnatal brain development, when neuronal differentiation, synapse formation, and circuit refinement occur rapidly. However, the specific mechanisms may vary by PFAS chain length, functional group, and toxicokinetic properties. The class-specific patterns observed in our study—stronger inverse associations for several long-chain PFCAs and 8:2 Cl-PFESA, but unexpected positive associations for some shorter-chain or legacy PFAS—suggest that PFAS neurodevelopmental associations may not be uniform across compounds.

This study has several strengths. The prospective design and measurement of PFAS in cord blood support temporal ordering between prenatal exposure and 24-month neurodevelopmental assessment. Cord blood provides a fetal-proximal biomarker of exposure near delivery and may reduce exposure misclassification relative to relying only on maternal samples. The study included a relatively large sample size and evaluated neurodevelopment across five BSID-III domains. The PFAS panel included both legacy compounds and emerging substitutes, allowing evaluation of compounds such as 8:2 Cl-PFESA.

Several limitations should be considered. First, cord blood was collected at delivery and may not capture trimester-specific exposure windows throughout pregnancy. We also did not distinguish between arterial and venous cord blood, which may introduce variability in exposure measurements. Second, although we adjusted for multiple maternal, child, and socioeconomic factors, residual confounding cannot be excluded. Third, the BSID-III < 90 threshold was used to indicate developmental risk or below-average performance, not to diagnose clinical developmental delay. Fourth, although WQS regression was useful for exploring correlated PFAS exposure patterns, it assumes a common direction of association within each direction-constrained model. Given the opposite directions observed across individual PFAS congeners, both negative- and positive-direction WQS weights and effect estimates should be interpreted cautiously. Finally, the study was conducted in an urban Chinese birth cohort, and generalizability to populations with different exposure profiles, socioeconomic characteristics, or healthcare contexts may be limited.

## Conclusion

This prospective study from the Shanghai Birth Cohort suggests that prenatal PFAS exposure, assessed using umbilical cord blood, is associated with domain-specific neurodevelopmental outcomes at 24 months. Selected long-chain PFAS, particularly PFNA, PFUdA, and PFTrDA, were consistently associated with lower cognitive and language scores, and the emerging substitute 8:2 Cl-PFESA showed similar inverse associations. Negative-direction WQS regression further suggested inverse mixture associations with cognitive and language scores. Future studies should incorporate repeated exposure assessment, paired maternal-cord biomarkers, breast-milk and infant postnatal PFAS measurements, longer neurodevelopmental follow-up, diverse populations, and mechanistic investigations to further clarify sensitive windows, causal pathways, and long-term effects of PFAS.

## Supplementary Information

Supplementary Material 1.

## Data Availability

The datasets used and/or analyzed during the current study are available from the corresponding author upon reasonable request, subject to ethical approval and data transfer agreements. The data are not publicly available due to privacy and ethical restrictions.

## Declarations

During manuscript preparation, ChatGPT was used solely to assist with English language editing and improving readability of selected text. It was not used to generate data, conduct statistical analyses, create figures or tables, or draw scientific conclusions. All AI-assisted text was critically reviewed, verified, and revised by the authors, who take full responsibility for the manuscript.
