# Supplementary material for: In utero PFAS exposure measured in the umbilical cord blood and child neurodevelopmental outcomes at 24 months: a prospective birth cohort study
Source: Environ Health. 2026 Aug 11;25:84. doi: 10.1186/s12940-026-01327-2 (PMC13741525; doi:10.1186/s12940-026-01327-2)
Supplement: Supplementary file 1 [file 12940_2026_1327_MOESM1_ESM.docx]

## **Supplemental Materials**

**Title:** In utero PFAS Exposure Measured in the Umbilical Cord Blood and Child Neurodevelopmental Outcomes at 24 Months: A Prospective Birth Cohort Study

**Authors:** Yiming Li, Bingjie Wu, Shiyang Li, Zhicao Yuan, Liu Liu, Yun Huang, Lei Tu, Jun Zhang

**Affiliations:**

School of Public Health, Shanghai Jiao Tong University School of Medicine, 227 South Chongqing Road, Shanghai 200052, China

**Corresponding author:**
Jun Zhang,PhD

School of Public Health, Shanghai Jiao Tong University School of Medicine, 227 South Chongqing Road, Shanghai 200052, China

junjimzhang@sjtu.edu.cn

Contents

[Table S1. Detection Limits, Detection Rates, and Concentrations of Per- and Polyfluoroalkyl Substances (PFAS) (ng/mL) in Cord Blood Serum	4](#_Toc32386)

[Table S2. Analytical Parameters for Quantification of PFAS in Umbilical Cord Blood Serum	6](#_Toc8360)

[Table S3. Unadjusted linear regression coefficients (β) and 95% confidence intervals (CI) for BSID-III scores in relation to log10-transformed cord blood PFAS concentrations [per log10-unit (ng/mL) increase] (n=1,668)	8](#_Toc30308)

[Table S4. Adjusted Linear Regression Coefficients (β) and 95% Confidence Intervals for BSID-III Scores Associated with Quartiles of Cord Blood PFAS Concentrations in Single-Pollutant Models (n=1,668)	10](#_Toc29528)

[Table S5. Adjusted linear regression coefficients (β) and 95% confidence intervals (CI) for BSID-III scores in relation to log₁₀-transformed cord blood PFAS concentrations [per log₁₀-unit (ng/mL) increase] in single-pollutant models excluding PFAS values that were greater than three times the value of the 75th percentile. (n=1,668)	15](#_Toc10738)

[Table S6. Sensitivity analysis using reduced linear regression models excluding gestational age at delivery, delivery mode, and any breastfeeding duration	17](#_Toc17910)

[Table S7. Sensitivity analysis using reduced logistic regression models excluding gestational age at delivery, delivery mode, and any breastfeeding duration	19](#_Toc6624)

[Table S8. Positive-direction weighted quantile sum (WQS) regression results for PFAS mixtures and BSID-III outcomes	21](#_Toc12271)

[Table S9. Missingness and multiple-imputation specifications for covariates included in the analytic models	22](#_Toc23374)

[Figure S1. Flowchart of participant selection in the Shanghai Birth Cohort study, analyzing associations between prenatal PFAS exposure (as measured in umbilical cord blood) and 24-month neurodevelopmental outcomes.	24](#_Toc26557)

[Figure S2. Spearman’s Rank Correlation Matrix of PFAS Concentrations in Umbilical Cord Blood Serum	25](#_Toc20797)

[Figure S3. Associations between log](#_Toc30982)_[10](#_Toc30982)_[-transformed cord serum PFAS concentrations and BSID cognition scores in a restricted cubic spline model.	26](#_Toc30982)

[Figure S4. Associations between log](#_Toc25276)_[10](#_Toc25276)_[-transformed cord serum PFAS concentrations and BSID language scores in a restricted cubic spline model.	27](#_Toc25276)

[Figure S5. Associations between log](#_Toc6298)_[10](#_Toc6298)_[-transformed cord serum PFAS concentrations and BSID motor scores in a restricted cubic spline model.	28](#_Toc6298)

[Figure S6. Associations between log](#_Toc7022)_[10](#_Toc7022)_[-transformed cord serum PFAS concentrations and BSID social-emotional scores in a restricted cubic spline model.	29](#_Toc7022)

[Figure S7. Associations between log](#_Toc27992)_[10](#_Toc27992)_[-transformed cord serum PFAS concentrations and BSID adaptive behavior scores in a restricted cubic spline model.	30](#_Toc27992)

[Figure S8. Weighted quantile sum (WQS) regression weights under positive-direction constraint for PFAS mixtures and BSID-III outcomes	31](#_Toc20827)

[Figure S9. Weighted quantile sum (WQS) regression weights under negative-direction constraint for PFAS mixtures and BSID-III outcomes	32](#_Toc7491)

[Figure S10. Directed acyclic graph (DAG) for cord blood PFAS concentration and child neurodevelopment at 24 months.	33](#_Toc551)

**Table S1.** Detection Limits, Detection Rates, and Concentrations of Per- and Polyfluoroalkyl Substances (PFAS) (ng/mL) in Cord Blood Serum

| **Abbreviation** | **Chemical Name** | **Carbon Chain Length** | **LOD (ng/mL)** | **Detection Rate (%)** | **PFAS Concentration (ng/mL)** | | | | |
| --- | --- | --- | --- | --- | --- | --- | --- | --- | --- |
|  |  |  |  |  | **Min** | **Q1** | **Median** | **Q3** | **Max** |
| **4:2 FTS** | 4:2 Fluorotelomer sulfonate | 4 | 0.0008 | 26.14 | 0.001 | 0.001 | 0.001 | 0.001 | 0.013 |
| **6:2 FTS** | 6:2 Fluorotelomer sulfonate | 6 | 0.0030 | 29.86 | 0.002 | 0.002 | 0.002 | 0.004 | 0.294 |
| **8:2 FTS** | 8:2 Fluorotelomer sulfonate | 8 | 0.0038 | 9.53 | 0.003 | 0.003 | 0.003 | 0.003 | 0.069 |
| **6:2 Cl-PFESA** | 6:2 Chlorinated polyfluoroether sulfonate | 6 | 0.0008 | 100.00 | 0.217 | 1.352 | 2.097 | 3.231 | 21.177 |
| **8:2 Cl-PFESA** | 8:2 Chlorinated polyfluoroether sulfonate | 8 | 0.0004 | 94.24 | 0.000 | 0.020 | 0.037 | 0.068 | 2.265 |
| **FOSA** | Perfluorooctanesulfonamide | 8 | 0.0010 | 99.52 | 0.001 | 0.011 | 0.019 | 0.031 | 0.775 |
| **HFPO-DA** | Hexafluoropropylene oxide dimer acid | 6 | 0.0010 | 65.71 | 0.001 | 0.001 | 0.004 | 0.008 | 2.903 |
| **ADONA** | 4,8-Dioxa-3H-perfluorononanoic acid | 9 | 0.0003 | 23.38 | 0.000 | 0.000 | 0.000 | 0.000 | 0.011 |
| **N-ETFOSAA** | N-ethyl perfluorooctanesulfonamidoacetic acid | 8 | 0.0041 | 9.71 | 0.003 | 0.003 | 0.003 | 0.003 | 0.017 |
| **N-MEFOSAA** | N-methyl perfluorooctanesulfonamidoacetic acid | 8 | 0.0020 | 49.40 | 0.001 | 0.001 | 0.001 | 0.004 | 0.317 |
| **PFBA** | Perfluorobutanoic acid | 4 | 0.0025 | 99.88 | 0.002 | 0.065 | 0.086 | 0.120 | 2.355 |
| **PFBS** | Perfluorobutanesulfonate | 4 | 0.0050 | 95.38 | 0.004 | 0.039 | 0.064 | 0.102 | 27.149 |
| **PFDA** | Perfluorodecanoic acid | 10 | 0.0061 | 99.76 | 0.004 | 0.275 | 0.430 | 0.672 | 5.346 |
| **PFDoA** | Perfluorododecanoic acid | 12 | 0.0150 | 94.18 | 0.011 | 0.044 | 0.069 | 0.105 | 47.600 |
| **PFDS** | Perfluorodecanesulfonate | 10 | 0.0060 | 23.68 | 0.004 | 0.004 | 0.004 | 0.004 | 0.034 |
| **PFHpA** | Perfluoroheptanoic acid | 7 | 0.0021 | 93.59 | 0.001 | 0.044 | 0.086 | 0.149 | 1.668 |
| **PFHpS** | Perfluoroheptanesulfonate | 7 | 0.0007 | 100.00 | 0.002 | 0.026 | 0.037 | 0.050 | 0.334 |
| **PFHxA** | Perfluorohexanoic acid | 6 | 0.0017 | 75.36 | 0.001 | 0.002 | 0.007 | 0.015 | 0.926 |
| **n-PFHxS** | Perfluorohexanesulfonate (linear) | 6 | 0.0035 | 100.00 | 0.017 | 0.190 | 0.254 | 0.340 | 4.150 |
| **Br-PFHxS** | Brominated perfluorohexanesulfonate | 6 | 0.0095 | 83.15 | 0.007 | 0.012 | 0.018 | 0.029 | 0.689 |
| **PFNA** | Perfluorononanoic acid | 9 | 0.0052 | 100.00 | 0.102 | 0.469 | 0.660 | 0.967 | 6.906 |
| **PFNS** | Perfluorononanesulfonate | 9 | 0.0100 | 1.50 | 0.007 | 0.007 | 0.007 | 0.007 | 0.045 |
| **PFOA** | Perfluorooctanoic acid | 8 | 0.0045 | 100.00 | 0.277 | 4.641 | 6.242 | 8.365 | 46.024 |
| **n-PFOS** | Perfluorooctanesulfonate (linear) | 8 | 0.0035 | 99.94 | 0.002 | 1.323 | 1.971 | 2.881 | 31.866 |
| **6m-PFOS** | Perfluorooctanesulfonate (6-methyl) | 8 | 0.0050 | 100.00 | 0.013 | 0.108 | 0.166 | 0.247 | 5.556 |
| **3,4,5m-PFOS** | Perfluorooctanesulfonate (3,4,5-methyl) | 8 | 0.0086 | 99.70 | 0.006 | 0.238 | 0.357 | 0.514 | 4.860 |
| **1m-PFOS** | Perfluorooctanesulfonate (1-methyl) | 8 | 0.0012 | 100.00 | 0.007 | 0.075 | 0.106 | 0.152 | 0.762 |
| **PFPeA** | Perfluoropentanoic acid | 5 | 0.0017 | 49.22 | 0.001 | 0.001 | 0.001 | 0.008 | 1.674 |
| **PFPeS** | Perfluoropentanesulfonate | 5 | 0.0010 | 91.97 | 0.001 | 0.003 | 0.004 | 0.006 | 0.294 |
| **PFTeDA** | Perfluorotetradecanoic acid | 14 | 0.0090 | 87.47 | 0.006 | 0.025 | 0.062 | 0.117 | 3.239 |
| **PFTrDA** | Perfluorotridecanoic acid | 13 | 0.0100 | 99.94 | 0.007 | 0.195 | 0.289 | 0.423 | 1.997 |
| **PFUdA** | Perfluoroundecanoic acid | 11 | 0.0100 | 100.00 | 0.054 | 0.287 | 0.422 | 0.634 | 6.093 |

**Notes：**LOD, limit of detection; Values below LOD were imputed as LOD/√2; All concentrations reported with three significant digits.

**Table S2.** Analytical Parameters for Quantification of PFAS in Umbilical Cord Blood Serum

| **PARAMETER** | **SPECIFICATION** |
| --- | --- |
| **Instrumentation** |  |
| System | Agilent 1290 Infinity UPLC coupled to 6495C Triple Quadrupole MS |
| Ionization | Negative electrospray (ESI-) |
| **Columns** |  |
| Online Purification | ZORBAX Eclipse Plus C18 (4.6 × 50 mm, 5 μm) |
| Analytical | ZORBAX RRHD Eclipse Plus C18 (2.1 × 50 mm, 1.8 μm) |
| Guard | ZORBAX Eclipse Plus C18 (2.1 × 5 mm, 1.8 μm) |
| **Chromatographic Conditions** |  |
| Column Temperature | 40°C |
| Injection Volume | 5 μL |
| Mobile Phase A | 0.01 M ammonium acetate in Milli-Q H₂O |
| Mobile Phase B | HPLC-grade methanol |
| Flow Rate | 0.4 mL/min |
| **Gradient Program** |  |
| Time (min) | % B |
| 0 | 40 |
| 0.75 | 40 |
| 2.75 | 60 |
| 6.75 | 65 |
| 9.70 | 90 |
| 13.70 | 98 (hold to 15.0 min) |
| **Mass Spectrometric Parameters** |  |
| Capillary Voltage | -3,500 V |
| Sheath Gas Flow | 15 L/min (375°C) |
| Nebulizer Pressure | 35 psi |
| Drying Gas | 15 L/min (200°C) |
| Source Temperature | 300°C |

**Table S3.** Unadjusted linear regression coefficients (β) and 95% confidence intervals (CI) for BSID-III scores in relation to log10-transformed cord blood PFAS concentrations [per log10-unit (ng/mL) increase] (n=1,668)

| **PFAS** | **β (95% CI)** | | | | |
| --- | --- | --- | --- | --- | --- |
|  | **cognitive scores** | **language scores** | **motor scores** | **Social-emotional scores** | **Adaptive behavior scores** |
| **6:2 Cl-PFESA** | -0.10 (-1.80,1.60) | -0.54 (-1.74,0.65) | 0.74 (-0.38,1.86) | -0.75 (-2.10,0.61) | 0.65 (-0.84,2.14) |
| **8:2 Cl-PFESA** | **-2.04 (-2.75,-1.33)** | **-1.28 (-1.78,-0.78)** | **-0.55 (-1.02,-0.07)** | 0.24 (-0.33,0.81) | **0.91 (0.28,1.54)** |
| **FOSA** | 0.33 (-1.02,1.67) | 0.12 (-0.83,1.06) | 0.47 (-0.42,1.36) | -0.07 (-1.14,1.01) | 0.35 (-0.84,1.53) |
| **HFPO-DA** | 0.10 (-0.68,0.88) | -0.35 (-0.90,0.19) | 0.03 (-0.49,0.54) | **0.83 (0.21,1.44)** | 0.46 (-0.21,1.14) |
| **PFBA** | 1.33 (-0.54,3.19) | 0.25 (-1.05,1.56) | **1.42 (0.19,2.65)** | -0.76 (-2.27,0.75) | 1.57 (-0.10,3.24) |
| **PFBS** | -0.45 (-1.58,0.67) | -0.22 (-1.00,0.57) | 0.44 (-0.30,1.18) | 0.18 (-0.71,1.07) | 0.49 (-0.49,1.47) |
| **PFDA** | 0.39 (-0.91,1.69) | -0.18 (-1.09,0.73) | 0.65 (-0.21,1.51) | -0.04 (-1.08,1.00) | 0.22 (-0.92,1.36) |
| **PFDoA** | **-2.11 (-3.43,-0.80)** | **-1.41 (-2.33,-0.48)** | -0.51 (-1.38,0.36) | 0.40 (-0.65,1.45) | **1.41 (0.25,2.57)** |
| **PFHpA** | **1.06 (0.22,1.89)** | 0.31 (-0.27,0.90) | **0.64 (0.09,1.20)** | 0.28 (-0.39,0.95) | -0.07 (-0.81,0.66) |
| **PFHpS** | 1.88 (-0.23,4.00) | 0.19 (-1.29,1.68) | 0.84 (-0.56,2.24) | -0.04 (-1.72,1.65) | 1.58 (-0.28,3.43) |
| **PFHxA** | **2.22 (1.38,3.06)** | **1.17 (0.58,1.76)** | **1.37 (0.81,1.93)** | 0.61 (-0.07,1.28) | 0.36 (-0.39,1.11) |
| **PFHxS(Total)** | **4.11 (2.01,6.22)** | 0.90 (-0.58,2.38) | **2.03 (0.63,3.42)** | 0.28 (-1.41,1.98) | 1.31 (-0.57,3.18) |
| **PFNA** | **-7.06 (-8.98,-5.13)** | **-4.47 (-5.82,-3.11)** | **-1.78 (-3.07,-0.49)** | -0.88 (-2.44,0.69) | **2.15 (0.43,3.87)** |
| **PFOA** | **3.85 (1.51,6.19)** | 0.70 (-0.95,2.34) | 1.52 (-0.03,3.07) | -0.52 (-2.39,1.36) | 0.50 (-1.57,2.57) |
| **PFOS(Total)** | 0.61 (-1.28,2.50) | -0.18 (-1.50,1.15) | 0.88 (-0.37,2.12) | -0.15 (-1.67,1.36) | 1.41 (-0.25,3.07) |
| **PFPeS** | **-1.70 (-3.10,-0.30)** | **-1.95 (-2.93,-0.97)** | **-1.14 (-2.06,-0.21)** | 0.72 (-0.40,1.83) | 0.89 (-0.34,2.11) |
| **PFTeDA** | **-1.66 (-2.62,-0.70)** | **-0.92 (-1.59,-0.25)** | -0.27 (-0.90,0.37) | -0.40 (-1.17,0.36) | 0.17 (-0.68,1.01) |
| **PFTrDA** | **-4.30 (-6.11,-2.49)** | **-2.18 (-3.45,-0.90)** | -0.20 (-1.41,1.00) | -0.30 (-1.76,1.16) | 1.40 (-0.20,3.01) |
| **PFUdA** | **-5.85 (-7.59,-4.11)** | **-3.30 (-4.53,-2.08)** | **-1.19 (-2.35,-0.02)** | -0.55 (-1.97,0.86) | **1.86 (0.30,3.41)** |

**Notes:**

1. Table presents crude (unadjusted) associations from linear regression models.
2. BSID-III: Bayley Scales of Infant and Toddler Development, Third Edition.
3. Compound abbreviations (see Table S1 for full names).
4. Bold text indicates statistical significance (95% CI excluding 0)

**Table S4.** Adjusted Linear Regression Coefficients (β) and 95% Confidence Intervals for BSID-III Scores Associated with Quartiles of Cord Blood PFAS Concentrations in Single-Pollutant Models (n=1,668)

| **PFAS quartiles** | **β (95% CI)** | | | | |
| --- | --- | --- | --- | --- | --- |
|  | **cognitive scores** | **language scores** | **motor scores** | **Social-emotional scores** | **Adaptive behavior scores** |
| **6:2 Cl-PFESA** |  |  |  |  |  |
| Q1 | Reference | Reference | Reference | Reference | Reference |
| Q2 | -0.69 (-3.56,2.18) | -2.08 (-4.14,-0.02) | 0.32 (-1.69,2.33) | 1.03 (-1.43,3.50) | 2.52 (-0.16,5.21) |
| Q3 | -0.57 (-3.46,2.32) | 0.67 (-1.40,2.75) | 1.31 (-0.71,3.33) | 0.22 (-2.27,2.71) | 1.07 (-1.63,3.77) |
| Q4 | -1.98 (-4.89,0.93) | -2.03 (-4.12,0.06) | 0.55 (-1.49,2.59) | -1.37 (-3.87,1.13) | 1.99 (-0.73,4.71) |
| p-Trend | 0.22 | 0.33 | 0.42 | 0.22 | 0.31 |
| **8:2 Cl-PFESA** |  |  |  |  |  |
| Q1 | Reference | Reference | Reference | Reference | Reference |
| Q2 | -2.15 (-4.98,0.68) | **-3.16 (-5.21,-1.11)** | -0.06 (-2.07,1.94) | 3.18 (0.72,5.63) | 1.23 (-1.45,3.90) |
| Q3 | **-5.58 (-8.43,-2.73)** | **-3.79 (-5.85,-1.73)** | -1.94 (-3.96,0.08) | 2.44 (-0.04,4.92) | 1.79 (-0.92,4.49) |
| Q4 | **-8.83 (-11.69,-5.98)** | **-5.59 (-7.65,-3.53)** | -2.32 (-4.34,-0.30) | -0.53 (-3.02,1.95) | 3.22 (0.51,5.94) |
| p-Trend | <0.01 | <0.01 | 0.01 | 0.55 | 0.02 |
| **FOSA** |  |  |  |  |  |
| Q1 | Reference | Reference | Reference | Reference | Reference |
| Q2 | 2.78 (-0.09,5.64) | 1.11 (-0.95,3.18) | 1.91 (-0.10,3.91) | 1.83 (-0.64,4.29) | 0.72 (-1.96,3.41) |
| Q3 | 1.13 (-1.74,4.01) | 0.25 (-1.81,2.32) | 1.42 (-0.59,3.43) | 0.64 (-1.82,3.11) | -0.54 (-3.23,2.15) |
| Q4 | 0.77 (-2.10,3.64) | 0.62 (-1.45,2.68) | 0.80 (-1.21,2.80) | 0.16 (-2.31,2.64) | 1.14 (-1.57,3.84) |
| p-Trend | 0.88 | 0.76 | 0.55 | 0.87 | 0.62 |
| **HFPO-DA** |  |  |  |  |  |
| Q1 | Reference | Reference | Reference | Reference | Reference |
| Q2 | **6.39 (3.53,9.24)** | -0.65 (-2.71,1.42) | -0.66 (-2.67,1.35) | 1.43 (-1.05,3.90) | -2.73 (-5.44,-0.03) |
| Q3 | 2.72 (-0.13,5.56) | -1.69 (-3.74,0.37) | -0.93 (-2.93,1.07) | 2.59 (0.14,5.05) | -0.40 (-3.08,2.28) |
| Q4 | 1.45 (-1.41,4.31) | -2.42 (-4.48,-0.35) | -1.37 (-3.38,0.65) | 2.80 (0.33,5.27) | 0.80 (-1.89,3.48) |
| p-Trend | 0.86 | 0.01 | 0.18 | 0.02 | 0.29 |
| **PFBA** |  |  |  |  |  |
| Q1 | Reference | Reference | Reference | Reference | Reference |
| Q2 | 3.49 (0.62,6.36) | 1.64 (-0.43,3.71) | 1.32 (-0.69,3.33) | -1.73 (-4.20,0.74) | 2.75 (0.06,5.43) |
| Q3 | 1.44 (-1.43,4.31) | 0.71 (-1.36,2.78) | 1.46 (-0.55,3.47) | 0.84 (-1.62,3.31) | -0.42 (-3.10,2.25) |
| Q4 | 1.23 (-1.62,4.09) | 0.13 (-1.93,2.19) | 2.12 (0.12,4.12) | -0.92 (-3.39,1.54) | 3.09 (0.41,5.77) |
| p-Trend | 0.72 | 0.87 | 0.04 | 0.96 | 0.16 |
| **PFBS** |  |  |  |  |  |
| Q1 | Reference | Reference | Reference | Reference | Reference |
| Q2 | -3.34 (-6.19,-0.48) | -2.47 (-4.52,-0.42) | -1.59 (-3.59,0.40) | -0.04 (-2.50,2.43) | 2.43 (-0.25,5.11) |
| Q3 | 0.73 (-2.15,3.60) | 0.64 (-1.43,2.71) | 1.16 (-0.85,3.17) | 0.04 (-2.44,2.52) | -0.34 (-3.03,2.36) |
| Q4 | -0.16 (-3.02,2.70) | 0.03 (-2.03,2.09) | 1.90 (-0.10,3.90) | 0.44 (-2.03,2.91) | 1.13 (-1.55,3.82) |
| p-Trend | 0.44 | 0.34 | 0.01 | 0.73 | 0.87 |
| **PFDA** |  |  |  |  |  |
| Q1 | Reference | Reference | Reference | Reference | Reference |
| Q2 | 2.54 (-0.31,5.39) | 1.78 (-0.27,3.83) | 0.40 (-1.60,2.40) | 1.69 (-0.77,4.16) | 1.16 (-1.52,3.84) |
| Q3 | 1.36 (-1.50,4.22) | 1.50 (-0.56,3.55) | 2.20 (0.19,4.21) | 0.68 (-1.79,3.15) | -0.17 (-2.87,2.52) |
| Q4 | -3.03 (-5.89,-0.17) | -2.12 (-4.17,-0.06) | 0.09 (-1.92,2.09) | -0.80 (-3.27,1.67) | 2.01 (-0.69,4.71) |
| p-Trend | 0.03 | 0.05 | 0.53 | 0.40 | 0.28 |
| **PFDoA** |  |  |  |  |  |
| Q1 | Reference | Reference | Reference | Reference | Reference |
| Q2 | 0.97 (-1.88,3.82) | 0.17 (-1.89,2.24) | -0.47 (-2.48,1.54) | 0.85 (-1.63,3.33) | -1.99 (-4.67,0.69) |
| Q3 | -0.49 (-3.34,2.37) | -0.29 (-2.36,1.77) | 1.20 (-0.81,3.21) | 1.94 (-0.53,4.42) | 2.37 (-0.33,5.06) |
| Q4 | **-6.74 (-9.64,-3.85)** | -3.29 (-5.39,-1.19) | -1.90 (-3.95,0.14) | -0.20 (-2.73,2.32) | 1.95 (-0.80,4.69) |
| p-Trend | <0.01 | <0.01 | 0.23 | 0.89 | 0.02 |
| **PFHpA** |  |  |  |  |  |
| Q1 | Reference | Reference | Reference | Reference | Reference |
| Q2 | -0.52 (-3.37,2.34) | -1.38 (-3.44,0.68) | -1.55 (-3.54,0.44) | -0.44 (-2.91,2.03) | -0.48 (-3.17,2.21) |
| Q3 | -1.02 (-3.89,1.86) | -0.61 (-2.68,1.46) | -0.12 (-2.13,1.89) | 1.31 (-1.16,3.79) | 1.28 (-1.41,3.98) |
| Q4 | 2.98 (0.09,5.88) | 0.79 (-1.30,2.87) | 2.41 (0.40,4.43) | 1.07 (-1.42,3.56) | 1.03 (-1.68,3.74) |
| p-Trend | 0.07 | 0.35 | 0.01 | 0.21 | 0.27 |
| **PFHpS** |  |  |  |  |  |
| Q1 | Reference | Reference | Reference | Reference | Reference |
| Q2 | 0.83 (-2.05,3.70) | 1.27 (-0.80,3.34) | 1.40 (-0.61,3.41) | -1.92 (-4.39,0.56) | 1.27 (-1.43,3.96) |
| Q3 | 0.00 (-2.89,2.89) | 0.99 (-1.08,3.07) | 0.78 (-1.24,2.80) | -1.35 (-3.84,1.14) | -0.27 (-2.98,2.44) |
| Q4 | 0.21 (-2.72,3.14) | -0.63 (-2.73,1.48) | 0.22 (-1.83,2.27) | -1.11 (-3.63,1.42) | 2.32 (-0.43,5.07) |
| p-Trend | 0.97 | 0.53 | 0.99 | 0.50 | 0.23 |
| **PFHxA** |  |  |  |  |  |
| Q1 | Reference | Reference | Reference | Reference | Reference |
| Q2 | **4.33 (1.47,7.19)** | **3.27 (1.21,5.33)** | 2.03 (0.03,4.03) | 1.09 (-1.40,3.57) | -0.41 (-3.12,2.29) |
| Q3 | **5.69 (2.82,8.55)** | **4.12 (2.06,6.18)** | **3.64 (1.63,5.64)** | 2.62 (0.14,5.11) | -0.13 (-2.84,2.57) |
| Q4 | **6.11 (3.24,8.98)** | **3.52 (1.45,5.58)** | **4.50 (2.49,6.51)** | 1.97 (-0.51,4.45) | 1.49 (-1.22,4.20) |
| p-Trend | <0.01 | <0.01 | <0.01 | 0.06 | 0.28 |
| **PFHxS(Total)** |  |  |  |  |  |
| Q1 | Reference | Reference | Reference | Reference | Reference |
| Q2 | -1.70 (-4.59,1.20) | -1.74 (-3.82,0.34) | 0.06 (-1.97,2.09) | 0.96 (-1.54,3.45) | 1.82 (-0.90,4.54) |
| Q3 | -0.02 (-2.94,2.90) | -1.23 (-3.33,0.86) | 0.59 (-1.45,2.64) | 0.52 (-1.99,3.04) | 1.05 (-1.69,3.78) |
| Q4 | 1.03 (-1.92,3.98) | -1.05 (-3.17,1.08) | 1.15 (-0.92,3.22) | -0.23 (-2.78,2.32) | 1.87 (-0.91,4.64) |
| p-Trend | 0.30 | 0.46 | 0.23 | 0.77 | 0.29 |
| **PFNA** |  |  |  |  |  |
| Q1 | Reference | Reference | Reference | Reference | Reference |
| Q2 | -0.05 (-2.90,2.79) | 1.01 (-1.04,3.06) | 0.09 (-1.92,2.10) | 1.51 (-0.97,3.98) | 2.25 (-0.43,4.94) |
| Q3 | -1.75 (-4.59,1.08) | -0.62 (-2.66,1.42) | 1.00 (-1.00,3.01) | -0.46 (-2.93,2.01) | 2.45 (-0.25,5.14) |
| Q4 | **-8.22 (-11.08,-5.36)** | **-5.06 (-7.12,-3.00)** | -1.52 (-3.54,0.50) | -1.05 (-3.54,1.45) | 2.93 (0.21,5.65) |
| p-Trend | <0.01 | <0.01 | 0.27 | 0.21 | 0.04 |
| **PFOA** |  |  |  |  |  |
| Q1 | Reference | Reference | Reference | Reference | Reference |
| Q2 | 2.99 (0.12,5.85) | -0.07 (-2.14,1.99) | 1.18 (-0.83,3.19) | 2.52 (0.06,4.99) | 1.52 (-1.17,4.20) |
| Q3 | **3.83 (0.95,6.70)** | 2.05 (-0.02,4.12) | 2.35 (0.34,4.37) | 1.22 (-1.26,3.70) | 1.75 (-0.96,4.45) |
| Q4 | 3.26 (0.37,6.14) | -0.27 (-2.35,1.81) | 0.92 (-1.11,2.94) | 0.31 (-2.18,2.80) | 0.60 (-2.11,3.31) |
| p-Trend | 0.02 | 0.70 | 0.23 | 0.93 | 0.64 |
| **PFOS(Total)** |  |  |  |  |  |
| Q1 | Reference | Reference | Reference | Reference | Reference |
| Q2 | -1.11 (-3.97,1.75) | 0.73 (-1.33,2.79) | 1.52 (-0.48,3.53) | -0.01 (-2.48,2.45) | 0.20 (-2.48,2.88) |
| Q3 | -0.72 (-3.60,2.16) | 0.67 (-1.40,2.74) | 0.56 (-1.46,2.57) | 0.92 (-1.56,3.40) | 0.95 (-1.75,3.64) |
| Q4 | -2.03 (-4.94,0.87) | -1.32 (-3.40,0.77) | 0.78 (-1.25,2.81) | -1.73 (-4.23,0.77) | 2.12 (-0.61,4.86) |
| p-Trend | 0.22 | 0.24 | 0.67 | 0.30 | 0.11 |
| **PFPeS** |  |  |  |  |  |
| Q1 | Reference | Reference | Reference | Reference | Reference |
| Q2 | -2.34 (-5.21,0.52) | -1.42 (-3.48,0.64) | -1.30 (-3.31,0.71) | 1.52 (-0.95,3.99) | 2.39 (-0.30,5.08) |
| Q3 | -2.68 (-5.56,0.21) | -1.69 (-3.76,0.38) | -1.39 (-3.41,0.63) | 0.16 (-2.33,2.65) | 1.56 (-1.15,4.26) |
| Q4 | -3.19 (-6.10,-0.29) | **-4.03 (-6.11,-1.95)** | -1.86 (-3.89,0.17) | 1.58 (-0.92,4.08) | 1.28 (-1.44,4.00) |
| p-Trend | 0.03 | <0.01 | 0.09 | 0.41 | 0.50 |
| **PFTeDA** |  |  |  |  |  |
| Q1 | Reference | Reference | Reference | Reference | Reference |
| Q2 | 1.11 (-1.76,3.97) | 0.46 (-1.60,2.52) | 2.53 (0.52,4.53) | 2.40 (-0.07,4.86) | 0.69 (-2.00,3.38) |
| Q3 | -0.06 (-2.94,2.81) | -0.08 (-2.15,1.99) | 1.42 (-0.59,3.43) | 1.43 (-1.05,3.91) | 1.63 (-1.08,4.34) |
| Q4 | -3.21 (-6.09,-0.33) | -2.20 (-4.28,-0.13) | -0.47 (-2.48,1.55) | -0.87 (-3.35,1.61) | -0.08 (-2.79,2.63) |
| p-Trend | 0.02 | 0.03 | 0.44 | 0.37 | 0.88 |
| **PFTrDA** |  |  |  |  |  |
| Q1 | Reference | Reference | Reference | Reference | Reference |
| Q2 | -1.88 (-4.72,0.96) | 0.27 (-1.78,2.32) | 1.45 (-0.55,3.45) | 0.71 (-1.75,3.18) | 1.22 (-1.47,3.91) |
| Q3 | -3.50 (-6.37,-0.63) | -0.74 (-2.80,1.33) | 0.30 (-1.71,2.32) | 0.51 (-1.97,2.99) | 0.87 (-1.83,3.56) |
| Q4 | **-5.98 (-8.87,-3.09)** | -2.74 (-4.82,-0.66) | 0.34 (-1.69,2.37) | 0.72 (-1.78,3.23) | 1.32 (-1.40,4.05) |
| p-Trend | <0.01 | 0.01 | 0.98 | 0.63 | 0.41 |
| **PFUdA** |  |  |  |  |  |
| Q1 | Reference | Reference | Reference | Reference | Reference |
| Q2 | -0.97 (-3.81,1.86) | -1.23 (-3.28,0.82) | 0.34 (-1.67,2.34) | 0.79 (-1.68,3.26) | 2.05 (-0.64,4.73) |
| Q3 | **-3.89 (-6.74,-1.04)** | -1.19 (-3.25,0.87) | 1.08 (-0.94,3.10) | -0.08 (-2.56,2.40) | 0.72 (-1.98,3.41) |
| Q4 | **-8.50 (-11.37,-5.64)** | **-4.80 (-6.87,-2.73)** | -1.44 (-3.47,0.59) | -1.77 (-4.27,0.73) | 3.78 (1.05,6.50) |
| p-Trend | <0.01 | <0.01 | 0.27 | 0.13 | 0.02 |

**Notes:**

1. Values are β (95% CI) representing the difference in BSID-III score for each quartile compared with Q1 (reference). Models are covariate-adjusted as described in Methods.
2. Linear trend p-values (p-trend) were estimated by modeling quartile as an ordinal variable.
3. Boldface indicates associations that remained statistically significant after false discovery rate (FDR) correction (q < 0.05).
4. Abbreviations: BSID-III, Bayley Scales of Infant and Toddler Development–Third Edition;β = regression coefficient; CI = confidence interval; Full compound names are provided in Table S1.

**Table S5.** Adjusted linear regression coefficients (β) and 95% confidence intervals (CI) for BSID-III scores in relation to log₁₀-transformed cord blood PFAS concentrations [per log₁₀-unit (ng/mL) increase] in single-pollutant models excluding PFAS values that were greater than three times the value of the 75th percentile. (n=1,668)

| **PFAS** | **β (95% CI)** | | | | |
| --- | --- | --- | --- | --- | --- |
|  | **cognitive scores** | **language scores** | **motor scores** | **Social-emotional scores** | **Adaptive behavior scores** |
| **6:2 Cl-PFESA** | -0.83 (-2.50 ,0.84 ) | -0.88 (-2.08 ,0.32 ) | 0.49 (-0.67 ,1.65 ) | -1.01 (-2.44 ,0.43 ) | 0.80 (-0.76 ,2.37 ) |
| **8:2 Cl-PFESA** | **-1.91 (-2.62 ,-1.20 )** | **-1.24 (-1.75 ,-0.73 )** | -0.48 (-0.98 ,0.02 ) | 0.36 (-0.24 ,0.97 ) | **0.85 (0.18 ,1.51 )** |
| **FOSA** | 0.04 (-1.29 ,1.38 ) | 0.18 (-0.78 ,1.14 ) | 0.58 (-0.35 ,1.51 ) | -0.19 (-1.34 ,0.95 ) | 0.16 (-1.09 ,1.41 ) |
| **HFPO-DA** | -0.15 (-1.08 ,0.79 ) | **-0.79 (-1.46 ,-0.11 )** | -0.05 (-0.70 ,0.60 ) | **1.12 (0.32 ,1.92 )** | 0.62 (-0.25 ,1.49 ) |
| **PFBA** | 0.90 (-1.03 ,2.84 ) | 0.56 (-0.83 ,1.95 ) | **1.83 (0.48 ,3.17 )** | -1.03 (-2.72 ,0.65 ) | **2.36 (0.52 ,4.21 )** |
| **PFBS** | -0.36 (-1.49 ,0.76 ) | -0.15 (-0.96 ,0.66 ) | 0.35 (-0.43 ,1.14 ) | 0.09 (-0.88 ,1.05 ) | 0.07 (-0.98 ,1.13 ) |
| **PFDA** | 0.24 (-1.01 ,1.48 ) | -0.17 (-1.06 ,0.72 ) | 0.67 (-0.19 ,1.54 ) | 0.02 (-1.04 ,1.09 ) | 0.38 (-0.78 ,1.53 ) |
| **PFDoA** | **-2.36 (-3.79 ,-0.93 )** | **-1.67 (-2.70 ,-0.65 )** | -0.30 (-1.29 ,0.70 ) | 0.11 (-1.11 ,1.33 ) | **1.63 (0.30 ,2.97 )** |
| **PFHpA** | **1.02 (0.22 ,1.82 )** | 0.31 (-0.27 ,0.88 ) | 0.53 (-0.02 ,1.09 ) | 0.34 (-0.34 ,1.03 ) | -0.05 (-0.80 ,0.69 ) |
| **PFHpS** | -0.07 (-2.17 ,2.03 ) | -0.83 (-2.33 ,0.67 ) | 0.17 (-1.29 ,1.62 ) | -0.59 (-2.38 ,1.20 ) | 1.41 (-0.54 ,3.37 ) |
| **PFHxA** | **2.48 (1.56 ,3.40 )** | **1.14 (0.47 ,1.80 )** | **1.56 (0.91 ,2.20 )** | 0.76 (-0.03 ,1.55 ) | 0.36 (-0.50 ,1.23 ) |
| **PFHxS(Total)** | 1.54 (-0.81 ,3.88 ) | -1.40 (-3.08 ,0.27 ) | 0.43 (-1.20 ,2.06 ) | 0.14 (-1.86 ,2.15 ) | 1.38 (-0.81 ,3.56 ) |
| **PFNA** | **-5.55 (-7.41 ,-3.68 )** | **-3.69 (-5.02 ,-2.35 )** | -1.31 (-2.62 ,0.00 ) | -0.68 (-2.29 ,0.94 ) | **2.08 (0.32 ,3.84 )** |
| **PFOA** | **3.26 (1.00 ,5.53 )** | 0.34 (-1.29 ,1.97 ) | 1.08 (-0.50 ,2.66 ) | -0.70 (-2.64 ,1.24 ) | 0.29 (-1.83 ,2.41 ) |
| **PFOS(Total)** | -0.68 (-2.56 ,1.19 ) | -0.82 (-2.16 ,0.53 ) | 0.59 (-0.71 ,1.89 ) | -0.69 (-2.30 ,0.91 ) | 1.25 (-0.50 ,3.00 ) |
| **PFPeS** | -1.10 (-2.47 ,0.28 ) | **-1.74 (-2.72 ,-0.76 )** | **-0.98 (-1.94 ,-0.03 )** | 0.48 (-0.69 ,1.65 ) | 0.71 (-0.56 ,1.99 ) |
| **PFTeDA** | **-1.18 (-2.12 ,-0.25 )** | **-0.80 (-1.47 ,-0.13 )** | -0.09 (-0.75 ,0.56 ) | -0.20 (-1.00 ,0.60 ) | 0.24 (-0.63 ,1.11 ) |
| **PFTrDA** | **-4.17 (-5.92 ,-2.42 )** | **-2.34 (-3.60 ,-1.08 )** | -0.12 (-1.35 ,1.10 ) | -0.29 (-1.81 ,1.22 ) | 1.06 (-0.59 ,2.71 ) |
| **PFUdA** | **-5.42 (-7.13 ,-3.70 )** | **-3.22 (-4.46 ,-1.99 )** | -0.92 (-2.12 ,0.29 ) | -0.47 (-1.97 ,1.02 ) | **1.86 (0.23 ,3.48 )** |

**Notes:**

1. Single-pollutant models included one PFAS compound at a time, adjusted for maternal age, pre-pregnancy BMI, gestational age, parity, education, occupation, self-rated household economic status, child sex, delivery mode, and any breastfeeding duration.
2. PFAS concentrations were log₁₀-transformed.
3. BSID-III: Bayley Scales of Infant and Toddler Development, Third Edition.
4. FDR correction was applied across PFAS congeners within each outcome domain.
5. Full compound names provided in Table S1.

**Table S6.** Sensitivity analysis using reduced linear regression models excluding gestational age at delivery, delivery mode, and any breastfeeding duration

| **PFAS** | **Cognitive scores** | **Language scores** | **Motor scores** | **Social-emotional scores** | **Adaptive behavior scores** |
| --- | --- | --- | --- | --- | --- |
| 6:2 Cl-PFESA | -0.50 (-2.11, 1.10) | -0.60 (-1.75, 0.55) | 0.64 (-0.48, 1.76) | -0.75 (-2.12, 0.63) | 0.90 (-0.59, 2.39) |
| 8:2 Cl-PFESA | -1.83 (-2.50, -1.15)*** | -1.18 (-1.66, -0.70)*** | -0.48 (-0.95, -0.01) | 0.28 (-0.29, 0.86) | 0.87 (0.24, 1.49) |
| FOSA | 0.63 (-0.63, 1.90) | 0.30 (-0.60, 1.21) | 0.62 (-0.26, 1.49) | 0.01 (-1.06, 1.09) | 0.43 (-0.75, 1.60) |
| HFPO-DA | -0.08 (-0.81, 0.64) | -0.49 (-1.00, 0.03) | -0.06 (-0.56, 0.45) | 0.79 (0.17, 1.41) | 0.38 (-0.29, 1.05) |
| PFBA | 0.61 (-1.13, 2.36) | -0.12 (-1.37, 1.13) | 1.22 (0.01, 2.43) | -0.82 (-2.33, 0.69) | 1.65 (-0.00, 3.29) |
| PFBS | -0.00 (-1.06, 1.05) | 0.04 (-0.71, 0.79) | 0.57 (-0.16, 1.30) | 0.25 (-0.65, 1.14) | 0.53 (-0.44, 1.51) |
| PFDA | 0.44 (-0.78, 1.66) | -0.05 (-0.93, 0.82) | 0.71 (-0.14, 1.56) | 0.01 (-1.03, 1.06) | 0.35 (-0.77, 1.48) |
| PFDoA | -1.55 (-2.80, -0.30)* | -1.17 (-2.06, -0.27)* | -0.29 (-1.16, 0.59) | 0.52 (-0.55, 1.59) | 1.32 (0.16, 2.48) |
| PFHpA | 1.27 (0.49, 2.06)** | 0.44 (-0.12, 1.01) | 0.68 (0.14, 1.23) | 0.31 (-0.36, 0.98) | -0.03 (-0.76, 0.69) |
| PFHpS | 0.53 (-1.46, 2.53) | -0.41 (-1.84, 1.01) | 0.41 (-0.98, 1.79) | -0.23 (-1.93, 1.47) | 1.67 (-0.18, 3.52) |
| PFHxA | 1.61 (0.82, 2.41)*** | 0.85 (0.28, 1.42)* | 1.18 (0.62, 1.73)*** | 0.54 (-0.14, 1.22) | 0.42 (-0.32, 1.17) |
| PFHxS(Total) | 2.82 (0.83, 4.80)* | 0.22 (-1.20, 1.65) | 1.60 (0.22, 2.99) | 0.10 (-1.60, 1.81) | 1.29 (-0.57, 3.15) |
| PFNA | -5.28 (-7.12, -3.45)*** | -3.51 (-4.83, -2.19)*** | -1.23 (-2.52, 0.06) | -0.69 (-2.28, 0.90) | 2.05 (0.33, 3.77) |
| PFOA | 3.85 (1.66, 6.05)** | 0.70 (-0.88, 2.27) | 1.44 (-0.09, 2.97) | -0.59 (-2.47, 1.28) | 0.42 (-1.63, 2.47) |
| PFOS(Total) | -0.05 (-1.83, 1.73) | -0.39 (-1.67, 0.88) | 0.68 (-0.56, 1.92) | -0.22 (-1.74, 1.30) | 1.52 (-0.14, 3.17) |
| PFPeS | -1.08 (-2.40, 0.24) | -1.67 (-2.60, -0.73)** | -0.93 (-1.84, -0.01) | 0.79 (-0.33, 1.90) | 0.72 (-0.50, 1.93) |
| PFTeDA | -1.16 (-2.06, -0.26)* | -0.64 (-1.28, 0.01) | -0.08 (-0.70, 0.55) | -0.33 (-1.09, 0.44) | 0.16 (-0.67, 1.00) |
| PFTrDA | -3.96 (-5.67, -2.24)*** | -2.21 (-3.44, -0.97)** | -0.09 (-1.30, 1.11) | -0.31 (-1.79, 1.17) | 0.92 (-0.69, 2.53) |
| PFUdA | -4.94 (-6.60, -3.28)*** | -2.88 (-4.08, -1.69)*** | -0.87 (-2.03, 0.30) | -0.43 (-1.87, 1.00) | 1.67 (0.11, 3.23) |

**Notes:** Single-pollutant reduced covariate models included one PFAS compound at a time, with log10-transformed concentrations [per log10-unit (ng/mL) increase]. Models were adjusted for maternal age, pre-pregnancy BMI, parity, maternal education, maternal occupation, self-rated household economic status, and child sex; gestational age at delivery, delivery mode, and any breastfeeding duration were excluded. Values are β (95% CI). Bold or starred values indicate associations that remained statistically significant after FDR correction across PFAS congeners within each outcome domain; * q<0.05, ** q<0.01, *** q<0.001. BSID-III, Bayley Scales of Infant and Toddler Development, Third Edition; CI, confidence interval.

**Table S7**. Sensitivity analysis using reduced logistic regression models excluding gestational age at delivery, delivery mode, and any breastfeeding duration

| **PFAS** | **Cognitive developmental risk** | **Language developmental risk** | **Motor developmental risk** | **Social-emotional developmental risk** | **Adaptive developmental risk** |
| --- | --- | --- | --- | --- | --- |
| 6:2 Cl-PFESA | 1.20 (0.90, 1.60) | 1.11 (0.93, 1.31) | 0.83 (0.60, 1.15) | 1.10 (0.90, 1.34) | 0.87 (0.72, 1.05) |
| 8:2 Cl-PFESA | 1.26 (1.09, 1.46)* | 1.18 (1.09, 1.28)*** | 1.11 (0.96, 1.29) | 0.98 (0.91, 1.07) | 0.90 (0.84, 0.97) |
| FOSA | 0.86 (0.70, 1.07) | 0.98 (0.86, 1.12) | 0.77 (0.62, 0.98) | 0.98 (0.84, 1.14) | 0.98 (0.85, 1.13) |
| HFPO-DA | 1.10 (0.96, 1.25) | 1.09 (1.01, 1.18) | 1.06 (0.92, 1.22) | 0.96 (0.88, 1.06) | 0.93 (0.86, 1.02) |
| PFBA | 0.81 (0.59, 1.12) | 1.01 (0.84, 1.22) | 1.00 (0.71, 1.42) | 1.01 (0.80, 1.26) | 0.79 (0.64, 0.97) |
| PFBS | 1.05 (0.87, 1.29) | 1.01 (0.91, 1.13) | 0.98 (0.80, 1.22) | 1.00 (0.87, 1.14) | 0.90 (0.80, 1.01) |
| PFDA | 0.79 (0.66, 0.96) | 1.03 (0.90, 1.17) | 0.73 (0.60, 0.91) | 0.93 (0.80, 1.08) | 0.91 (0.79, 1.05) |
| PFDoA | 0.98 (0.80, 1.19) | 1.23 (1.07, 1.41)* | 0.96 (0.76, 1.21) | 0.86 (0.73, 1.00) | 0.78 (0.67, 0.90)* |
| PFHpA | 0.84 (0.75, 0.96)* | 0.95 (0.88, 1.03) | 0.89 (0.77, 1.03) | 1.01 (0.92, 1.12) | 0.93 (0.86, 1.02) |
| PFHpS | 1.19 (0.84, 1.70) | 1.19 (0.97, 1.48) | 0.98 (0.66, 1.45) | 1.03 (0.80, 1.32) | 0.76 (0.60, 0.95) |
| PFHxA | 0.91 (0.79, 1.04) | 0.92 (0.85, 1.01) | 0.88 (0.75, 1.03) | 0.99 (0.90, 1.09) | 0.94 (0.85, 1.03) |
| PFHxS(Total) | 0.84 (0.58, 1.21) | 1.16 (0.94, 1.43) | 0.77 (0.50, 1.15) | 0.99 (0.77, 1.27) | 0.79 (0.62, 1.00) |
| PFNA | 1.95 (1.41, 2.71)** | 1.75 (1.43, 2.14)*** | 1.09 (0.76, 1.55) | 1.00 (0.80, 1.27) | 0.77 (0.62, 0.96) |
| PFOA | 0.79 (0.53, 1.17) | 1.02 (0.81, 1.29) | 0.77 (0.50, 1.18) | 0.98 (0.74, 1.29) | 0.87 (0.67, 1.12) |
| PFOS(Total) | 1.14 (0.83, 1.56) | 1.13 (0.94, 1.36) | 0.82 (0.57, 1.16) | 1.03 (0.82, 1.28) | 0.83 (0.67, 1.02) |
| PFPeS | 1.18 (0.94, 1.50) | 1.37 (1.19, 1.59)*** | 1.20 (0.92, 1.56) | 0.93 (0.79, 1.09) | 0.93 (0.80, 1.08) |
| PFTeDA | 1.03 (0.88, 1.20) | 1.01 (0.92, 1.11) | 1.00 (0.84, 1.19) | 1.10 (0.98, 1.23) | 1.00 (0.90, 1.10) |
| PFTrDA | 1.53 (1.12, 2.11)* | 1.42 (1.18, 1.72)*** | 0.86 (0.62, 1.20) | 0.99 (0.80, 1.23) | 0.88 (0.72, 1.08) |
| PFUdA | 1.72 (1.27, 2.32)** | 1.58 (1.31, 1.90)*** | 1.01 (0.73, 1.40) | 0.99 (0.80, 1.22) | 0.79 (0.65, 0.97) |

**Notes:** Single-pollutant reduced covariate logistic models included one PFAS compound at a time, with log10-transformed concentrations [per log10-unit (ng/mL) increase]. Developmental risk was defined as BSID-III composite score <90; the reference group was BSID-III score ≥90. Models were adjusted for maternal age, pre-pregnancy BMI, parity, maternal education, self-rated household economic status, and child sex; gestational age at delivery, delivery mode, any breastfeeding duration, and maternal occupation were excluded. Maternal occupation was omitted because sparse categories led to model instability. Values are OR (95% CI). Bold or starred values indicate associations that remained statistically significant after FDR correction across PFAS congeners within each outcome domain; * q<0.05, ** q<0.01, *** q<0.001. BSID-III, Bayley Scales of Infant and Toddler Development, Third Edition; CI, confidence interval; OR, odds ratio.

**Table S8**. Positive-direction weighted quantile sum (WQS) regression results for PFAS mixtures and BSID-III outcomes

| **Method** | **Outcome domain** | **Effect estimate (β) (95% CI)** | **P-value** |
| --- | --- | --- | --- |
| WQS regression (positive-direction constraint) | Cognitive scores | 2.27 (0.29, 4.24) | 0.02 |
|  | Language scores | 0.33 (-1.16, 1.82) | 0.66 |
|  | Motor scores | 1.64 (0.28, 3.00) | 0.02 |
|  | Social-emotional scores | -1.16 (-3.17, 0.86) | 0.26 |
|  | Adaptive behavior scores | 1.27 (-0.97, 3.52) | 0.27 |

**Notes:** WQS regression was conducted as a supplementary positive-direction sensitivity analysis. All models were adjusted for maternal age, pre-pregnancy BMI, parity, maternal education, maternal occupation, self-rated household economic status, gestational age at delivery, child sex, delivery mode, and any breastfeeding duration. Because WQS regression is directionally constrained, these results were interpreted cautiously and considered secondary to the main single-pollutant findings and the negative-direction WQS model shown in the main manuscript.

**Table S9**. Missingness and multiple-imputation specifications for covariates included in the analytic models

| **Covariate** | **Missing n (%)** | **Variable type** | **Imputation method** | **Role in analytic models** |
| --- | --- | --- | --- | --- |
| Maternal age | 0 (0.0) | Continuous | Complete; predictor only | Covariate |
| Pre-pregnancy BMI | 0 (0.0) | Continuous | Complete; predictor only | Covariate |
| Parity | 229 (13.7) | Binary categorical | Logistic regression | Covariate |
| Maternal education | 228 (13.7) | Binary categorical | Logistic regression | Covariate |
| Maternal occupation | 546 (32.7) | Unordered categorical | Multinomial logistic regression | Covariate |
| Self-rated household economic status | 274 (16.4) | Ordered categorical | Proportional odds model | Covariate |
| Gestational age at delivery | 0 (0.0) | Continuous | Complete; predictor only | Fully adjusted model; excluded in reduced model |
| Child sex | 22 (1.3) | Binary categorical | Logistic regression | Covariate |
| Delivery mode | 72 (4.3) | Unordered categorical | Multinomial logistic regression | Fully adjusted model; excluded in reduced model |
| Any breastfeeding duration | 92 (5.5) | Ordered categorical | Proportional odds model | Fully adjusted model; excluded in reduced model |

**Notes:** Missingness was assessed before multiple imputation. PFAS concentrations and BSID-III scores were included as predictors but were not imputed. PFAS values below LOD were replaced with LOD/√2. BMI, body mass index; BSID-III, Bayley Scales of Infant and Toddler Development, Third Edition; LOD, limit of detection; PFAS, per- and polyfluoroalkyl substances.

**Figure S1.** Flowchart of participant selection in the Shanghai Birth Cohort study, analyzing associations between prenatal PFAS exposure (as measured in umbilical cord blood) and 24-month neurodevelopmental outcomes.

**Figure S2.** Spearman’s Rank Correlation Matrix of PFAS Concentrations in Umbilical Cord Blood Serum

**Notes:** Spearman’s rank correlation coefficients are shown, ranging from -1 to 1. Color intensity reflects the strength of positive correlations, with darker blue indicating stronger associations. 'X' denotes correlations not calculated due to low detection rates or insufficient quantifiable concentrations for at least one PFAS congener. Full names of PFAS congeners are provided in Table S1.

**Figure S3.** Associations between log_10_-transformed cord serum PFAS concentrations and BSID cognition scores in a restricted cubic spline model.

**Notes:** The solid lines (red) represent the regression coefficient, and the shaded regions (pink) correspond to the 95% confidence intervals of the regression coefficient. Knots were fixed at the 5th, 27.5th, 50th, 72.5th, and 95th percentiles of the log10-transformed PFAS concentrations. The models were adjusted for maternal age, pre-pregnancy body mass index (BMI), gestational age at delivery, parity, maternal education, maternal occupation, self-rated household economic status, child sex, delivery mode, and any breastfeeding duration.

**Figure S4.** Associations between log_10_-transformed cord serum PFAS concentrations and BSID language scores in a restricted cubic spline model.

**Notes:** The solid lines (red) represent the regression coefficient, and the shaded regions (pink) correspond to the 95% confidence intervals of the regression coefficient. Knots were fixed at the 5th, 27.5th, 50th, 72.5th, and 95th percentiles of the log10-transformed PFAS concentrations. The models were adjusted for maternal age, pre-pregnancy body mass index (BMI), gestational age at delivery, parity, maternal education, maternal occupation, self-rated household economic status, child sex, delivery mode, and any breastfeeding duration.

**Figure S5.** Associations between log_10_-transformed cord serum PFAS concentrations and BSID motor scores in a restricted cubic spline model.

**Notes:** The solid lines (red) represent the regression coefficient, and the shaded regions (pink) correspond to the 95% confidence intervals of the regression coefficient. Knots were fixed at the 5th, 27.5th, 50th, 72.5th, and 95th percentiles of the log10-transformed PFAS concentrations. The models were adjusted for maternal age, pre-pregnancy body mass index (BMI), gestational age at delivery, parity, maternal education, maternal occupation, self-rated household economic status, child sex, delivery mode, and any breastfeeding duration.

**Figure S6.** Associations between log_10_-transformed cord serum PFAS concentrations and BSID social-emotional scores in a restricted cubic spline model.

**Notes:** The solid lines (red) represent the regression coefficient, and the shaded regions (pink) correspond to the 95% confidence intervals of the regression coefficient. Knots were fixed at the 5th, 27.5th, 50th, 72.5th, and 95th percentiles of the log10-transformed PFAS concentrations. The models were adjusted for maternal age, pre-pregnancy body mass index (BMI), gestational age at delivery, parity, maternal education, maternal occupation, self-rated household economic status, child sex, delivery mode, and any breastfeeding duration.

**Figure S7.** Associations between log_10_-transformed cord serum PFAS concentrations and BSID adaptive behavior scores in a restricted cubic spline model.

**Notes:** The solid lines (red) represent the regression coefficient, and the shaded regions (pink) correspond to the 95% confidence intervals of the regression coefficient. Knots were fixed at the 5th, 27.5th, 50th, 72.5th, and 95th percentiles of the log10-transformed PFAS concentrations. The models were adjusted for maternal age, pre-pregnancy body mass index (BMI), gestational age at delivery, parity, maternal education, maternal occupation, self-rated household economic status, child sex, delivery mode, and any breastfeeding duration.

**Figure S8.** Weighted quantile sum (WQS) regression weights under positive-direction constraint for PFAS mixtures and BSID-III outcomes

**Notes:** In this supplementary positive-direction WQS analysis, the WQS index was constrained to favor positive associations, corresponding to higher developmental scores. Red dashed line marks the 1/M threshold; PFAS exceeding this line are considered dominant contributors. Separate panels depict weights for cognitive, language, motor, social-emotional, and adaptive behavior scores. Models adjust for covariates as detailed in Methods.

**Figure S9.** Weighted quantile sum (WQS) regression weights under negative-direction constraint for PFAS mixtures and BSID-III outcomes

**Notes:** Each panel shows weights assigned to PFAS in constructing the WQS index constrained toward adverse effects (lower developmental scores). The red dashed line indicates the 1/M threshold (M = 19; ≈0.053), with bars exceeding this threshold denoting the most influential contributors to the mixture effect. Panels correspond to cognitive, language, motor, social-emotional, and adaptive behavior scores. Models adjust for covariates as detailed in Methods.

**Figure S10.** Directed acyclic graph (DAG) for cord blood PFAS concentration and child neurodevelopment at 24 months.

**Notes:** Blue nodes indicate a priori confounders selected based on prior literature and the study DAG framework. For graphical clarity, direct paths from the blue confounder nodes to the outcome are summarized here rather than drawn individually. In the full causal framework, these variables were assumed to affect both cord blood PFAS concentration and child neurodevelopment and therefore were considered potential confounders of the exposure-outcome association. Orange nodes indicate perinatal or postnatal variables that may have complex roles in the exposure-outcome relationship and were evaluated in sensitivity analyses. Gestational age at delivery, delivery mode, and any breastfeeding duration were included in the fully adjusted models for comparability with prior PFAS-neurodevelopment studies. However, because these variables may also be conceptualized as potential mediators, downstream factors, or postnatal determinants of neurodevelopment, reduced-covariate models excluding these variables were additionally conducted. Any breastfeeding duration was treated with particular caution because it may reflect both beneficial postnatal nutrition and bonding and a potential route of postnatal PFAS transfer through breast milk.
